# A Systematic Methodology for the Identification of the Chemical Composition of the Mongolian Drug Erdun-Uril Compound Utilizing UHPLC-Q-Exactive Orbitrap Mass Spectrometry

**DOI:** 10.3390/molecules29184349

**Published:** 2024-09-13

**Authors:** Yanghui Huo, Kailin Li, Suyu Yang, Bo Yi, Zhihua Chai, Lingxuan Fan, Liangyin Shu, Bowen Gao, Huanting Li, Wei Cai

**Affiliations:** 1 School of Pharmacy, Baotou Medical College, Baotou 014040, China; hyanghui818@163.com (Y.H.);; 2 School of Pharmaceutical Sciences, Hunan University of Medicine, Huaihua 418000, China; 3 School of Pharmacy, Shandong Second Medical University, Weifang 261000, China

**Keywords:** UHPLC-Q-Exactive Orbitrap MS, identification, Erdun-Uril, Mongolian medicine

## Abstract

The traditional Mongolian medicine Erdun-Uril is a conventional combination of 29 herbs commonly used for the treatment of cerebrovascular ailments. It has the effects of reducing inflammation, counteracting oxidative stress, and averting strokes caused by persistent cerebral hypoperfusion. Prior research on Erdun-Uril has predominantly concentrated on its pharmacodynamics and mechanism of action; however, there has been a lack of systematic and comprehensive investigation into its chemical constituents. Therefore, it is crucial to establish an efficient and rapid method for evaluating the chemical constituents of Erdun-Uril. In this study, Erdun-Uril was investigated using UHPLC-Q-Exactive Orbitrap MS combined with parallel reaction monitoring for the first time. Eventually, a total of 237 compounds, including 76 flavonoids, 68 phenolic compounds, 19 alkaloids, 7 amino acids, etc., were identified based on the chromatographic retention time, bibliography data, MS/MS^2^ information, neutral loss fragments (NLFs), and diagnostic fragment ions (DFIs). And of these, 225 were reported for the first time in this study. This new discovery of these complex components would provide a reliable theoretical basis for the development of pharmacodynamics and quality standards of the Mongolian medicine Erdun-Uril.

## 1. Introduction

Erdun-Uril, also known as the Zhenbao pill or Sobud Uril, meaning “precious and miraculous pill”, originated from ancient Mongolian medicine books, and is recorded in the Mongolian medical classics Manag Mudigpo Yewa (Secret Supplement) and Mongolian Medical Jinkui [1,2]. It mostly consists of 29 herbs, including *Glycyrrhiza uralensis Fisch*, *Aquilaria* spp., *Dalbergia odorifera* T. Chen, *Gardenia jasminoides* J. Ellis, *Carthamus tinctorius* L., *Piper longum* L., *Syringa oblata Lindl.*, and *Lygodium japonicum (Thunb.)* S., etc. [2,3]. Previous investigations [3,4,5,6] have shown that Erdun-Uril has many pharmacological effects, including antioxidant properties, vasodilation, and modulation of blood lipid levels, which have been widely used for the treatment of cerebrovascular ailments in clinical applications, such as cerebral hemorrhage, stroke, epilepsy, Alzheimer’s disease, and Parkinson’s disease [3,4,5,6,7]. Among them, *Gardenia jasminoides Ellis*, *Cassiae Semen*, and *Terminalia chebula Retz*. have all been reported to have lipid-regulating effects [8], whereas *Gardenia jasminoides Ellis*, along with drugs such as *Syringa oblata Lindl*. and *Dalbergiae Odoriferae*, has been shown to have antiplatelet coagulation and thrombosis-reducing effects [9], and *Radix Aucklandiae* and *Inula helenium L.* have been reported to have anti-atherosclerotic effects. Both *Carthamus tinctorius L.* and *Glycyrrhiza uralensis Fisch*. can synergistically improve energy metabolism as well as anti-inflammatory and analgesic effects in rats with cold coagulation and blood stasis [10]. At the same time, the currently available drug safety evaluation shows that the relevant biochemical indexes are still within the reasonable range when ten times the clinical dose of Erdun-Uril is given to rats, which indicates that the drug has a favorable safety profile [11]. However, compared with the research of pharmacological effects and clinical applications, there are few reports on the chemical composition of Erdun-Uril. Therefore, it is worth establishing a rapid and comprehensive analytical strategy to characterize its chemical composition.

UHPLC-Q-Exactive Orbitrap MS technology has high-throughput scanning and multiple detection capabilities and has been widely used to analyze complex systems [12,13,14]. The complete MS/dd MS^2^ mode typically acquires sample data by first detecting the primary precursor ion, then performing secondary mass spectrometry triggered by the top three higher intensities. However, the constraints of the analytical process make it challenging to extract and analyze minor components [15,16,17]. Recent research shows that the parallel reaction monitoring (PRM) mode enhances sensitivity compared to the full MS scan mode. [18,19,20]. Therefore, the aim of our study was to develop an analytical method based on UHPLC Q-Exactive Orbitrap MS technology combined with parallel reaction monitoring, enabling diagnostic fragment ions (DFIs) and neutral loss fragments (NLFs) to identify the major chemicals in Erdun-Uril.

During this procedure, a total of 237 components were detected using UHPLC-Q-Exactive Orbitrap mass spectrometry (Thermo Fisher Scientific Co., Ltd., Waltham, MA, USA), of which 225 were first reported in Erdun-Uril. These findings might assist us in conducting quality control of Erdun-Uril in future clinical trials and provide foundations for crucial research on the pharmacodynamic components of Erdun-Uril.

## 2. Results and Discussion

### 2.1. Analytical Strategy

The aim of this study was to methodically identify the chemical constituents of Erdun-Uril. Consequently, a highly effective approach utilizing UHPLC-Q-Exactive Orbitrap MS combined with parallel reaction monitoring (PRM) was developed. Initially, the chemicals in Erdun-Uril were extracted and enriched by applying ultrasonic extraction with 70% methanol. Furthermore, the sample was injected to the UHPLC-Q-Exactive Orbitrap mass spectrometer in order to acquire high-resolution MS data for Erdun-Uril. Additionally, the UHPLC-Q-Exactive Orbitrap MS paired with PRM scanning was used to gather the MS^2^ data for the trace components in Erdun-Uril. Finally, the potential chemicals were determined by comparing them with standards, summary DFIs, and neutral loss, as well as by referencing the literature.

### 2.2. Establishment of Diagnostic Fragment Ions (DFIs) and Neutral Loss Fragments (NLFs)

DFIs and NLFs are both well-acknowledged screening methods that are used to identify the complex traditional Chinese medicine system composition based on the accurate ion mass and specific fragment information provided by the high-resolution mass spectrum and standard reference [21,22,23]. In contrast to the traditional way of identifying compounds derived from the literature, this approach can deduce the specific structure of a compound based on mass spectrometry information, which is suitable for identifying the multiple compounds contained in Erdun-Uril. As an example, flavonoids possess 2-phenylchromanone as their parent structure, which is also subdivided into flavonols, flavonoid glycosides, and isoflavonoids based on the different substituent groups on the parent nucleus. Therefore, the cleavage fragments of 2-phenylchromanone can be used as the DFIs of flavonoids, and the fragment ions with different substituents can be used as the basis for the identification of compounds. Some fragment ions are detected by the NLF method in the form of a fixed mass difference between the fragments; NLFs are also valuable for the identification of compounds.

**Figure 1:**
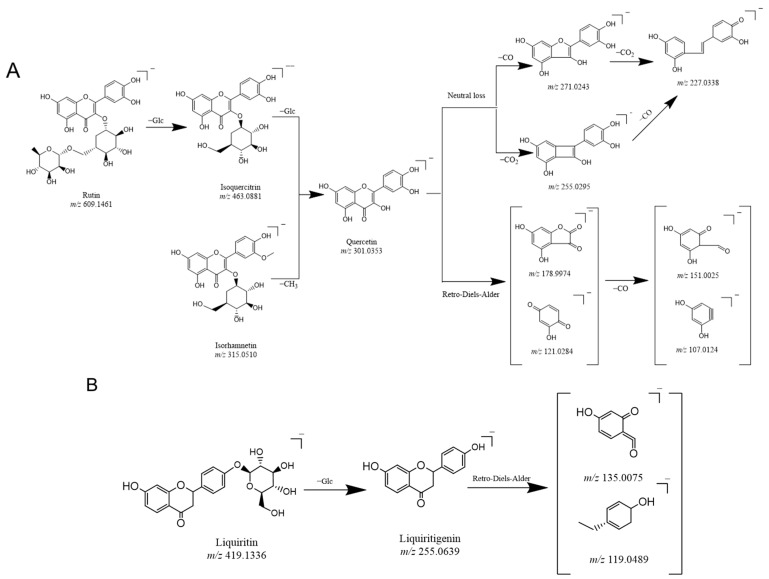

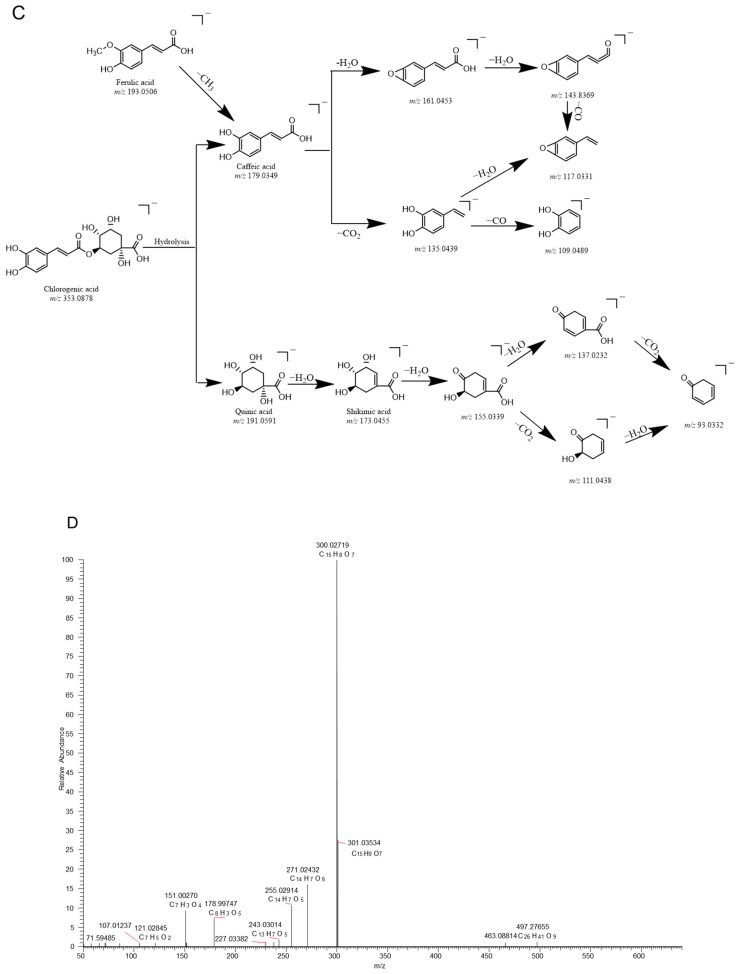

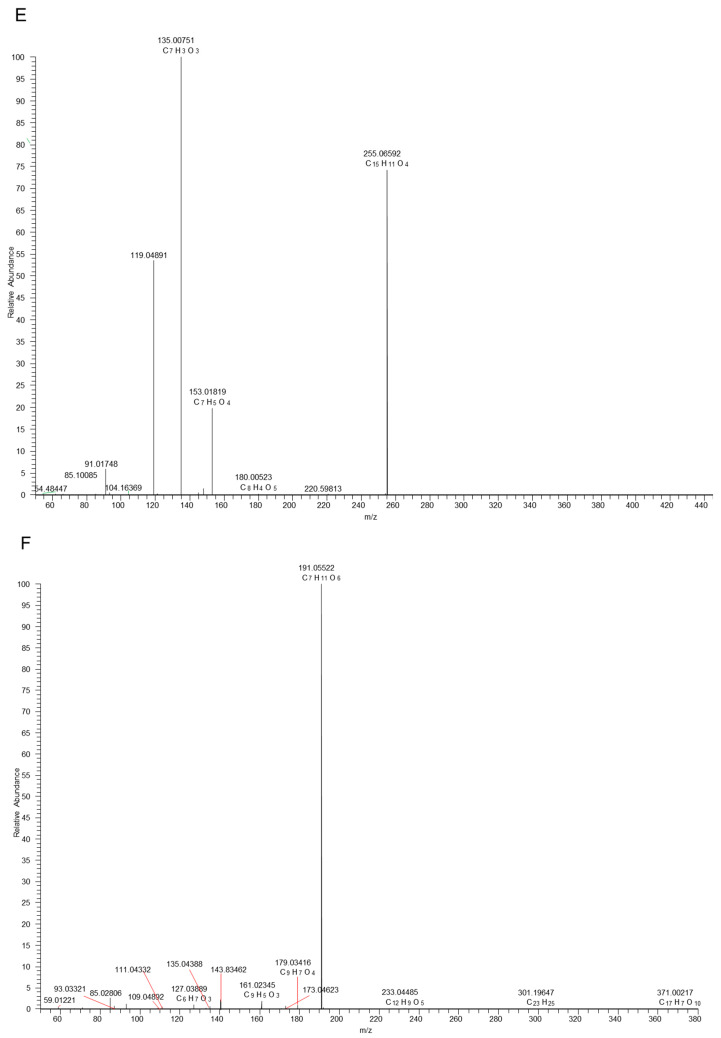
Proposed selected fragmentation pattern of components identified in Erdun-Uril flavonols (**A**), dihydroflavonoids (**B**), phenolic acids (**C**), and mass spectra of standards: Rutin (**D**), liquiritin (**E**), and chlorogenic acid (**F**).

In this study, the fragment ion patterns of flavonols, dihydroflavonoids, and phenolic acids were investigated using LC-MS/MS. The fragmentation pathway of flavonols and dihydroflavonoids is shown in Figure 1A–C. The same fragmentations were identified as 178.9974 (C_8_H_3_O_5_), 151.0025 (C_7_H_3_O_4_), 121.0281 (C_7_H_5_O_2_), and 107.0124 (C_6_H_3_O_2_), which could be regarded as the DFIs of flavonols. In addition, the fragments 135.0075 (C_7_H_4_O_3_) and 119.0489 (C_8_H_7_O) were displayed in the fragmentation pathway as the DFIs of dihydroflavonoids. Likewise, phenolic acids contain phenolic hydroxyl and carboxyl groups and easily lose H_2_O, CO_2_, and CO neutral groups during cracking. This was confirmed by the fragment ions obtained from the secondary mass spectra of several phenolic acid standard controls, all of which showed neutral loss and generated [M − H_2_O], [M − CO], and [M − CO_2_] fragments during cleavage.

### 2.3. Characterization of the Chemical Constitution in Erdun-Uril

**Figure 2:**
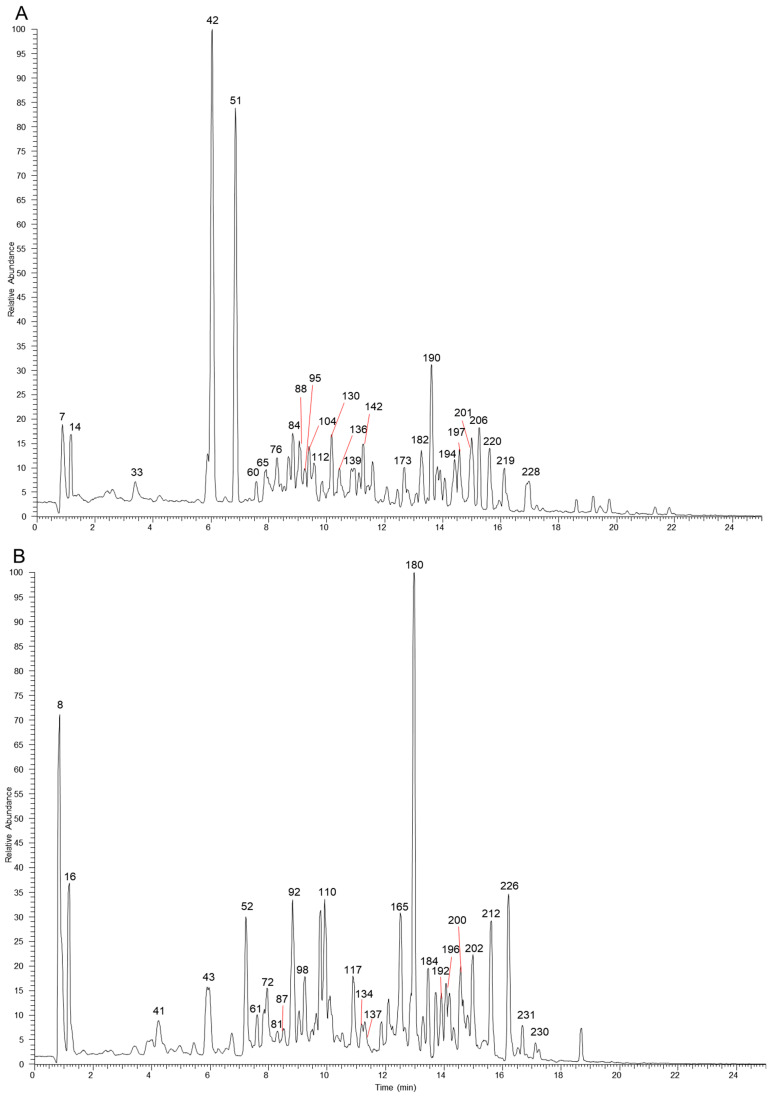

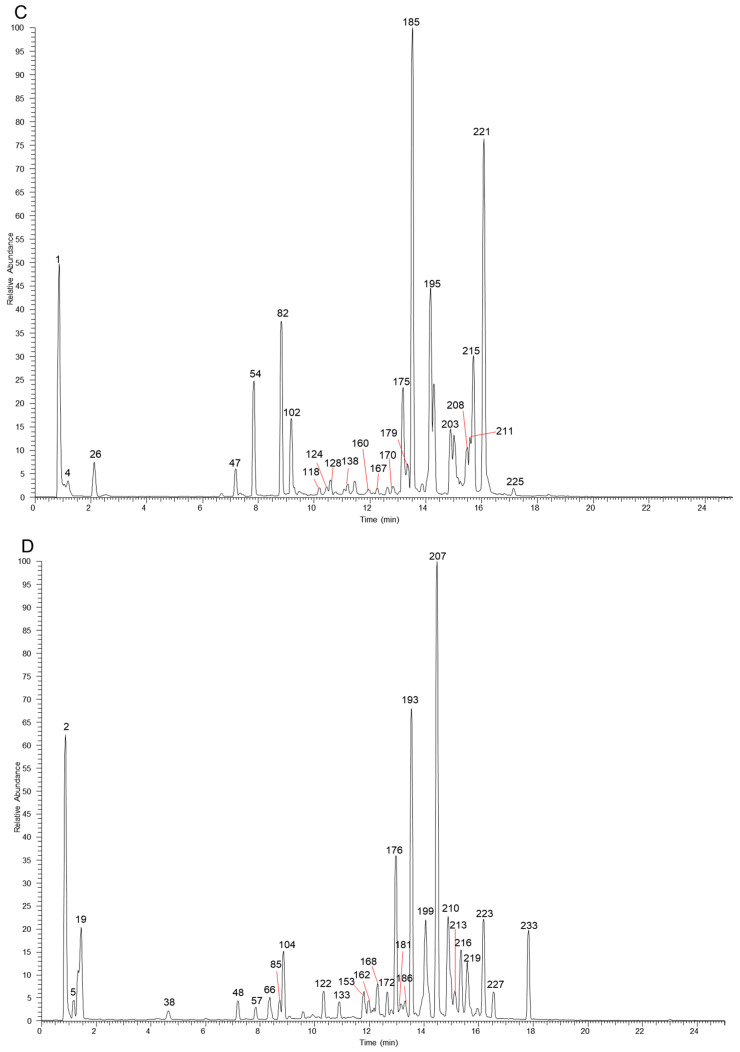
The high-resolution extracted ion chromatograms (EIC) in 10 ppm for multiple compounds in Erdun-Uril in positive mode. (**A**) *m/z* 124.0393, 165.0546, 127.0389, 355.1023, 291.0863, 249.1485, 565.1551, 193.0495, 573.1578, 595.1657, 371.1125, 493.0976, 301.0706, 303.0499, 507.1133, 271.06, 287.055, 355.1176, 345.1696, 455.3519, 367.1176, 265.1223, 325.1434, 165.091, 236.2008, 279.159; (**B**) *m/z* 130.0498, 268.104, 163.0389, 347.17, 225.0757, 153.0546, 183.0651, 479.082, 419.1336, 463.0871, 317.0291, 551.1759, 269.0444, 537.233, 855.4008, 179.0338, 135.0804, 353.1383, 355.154, 163.0753, 372.1805, 312.1594, 314.175, 293.1747, 300.2897, 325.141; (**C**) *m/z* 118.0862, 152.0566, 136.0617, 166.0862, 177.0546, 167.0704, 209.0808, 319.1176, 179.0702, 431.1336, 285.0757, 219.1743, 248.1281, 369.1332, 318.1335, 274.1437, 203.1794, 311.1277, 193.0859, 251.1066, 233.1536, 231.1379, 165.091, 330.2063, 340.1907, 342.2063, 179.1066, 252.2321; (**D**) *m/z* 116.0706, 182.0811, 132.1019, 114.0913, 551.1963, 118.0651, 169.1223, 257.0808, 147.044, 337.0837, 258.1488, 823.411, 272.1281, 267.1015, 391.2842, 327.159, 288.1594, 397.1586, 281.1172, 195.1015, 328.1907, 224.2008, 233.1536, 221.1899, 209.1172, 344.222, 384.2533.

**Figure 3:**
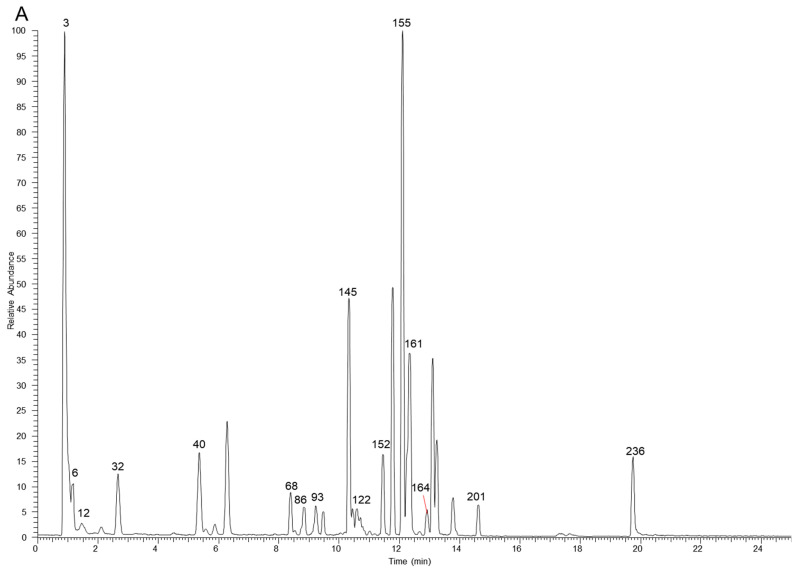

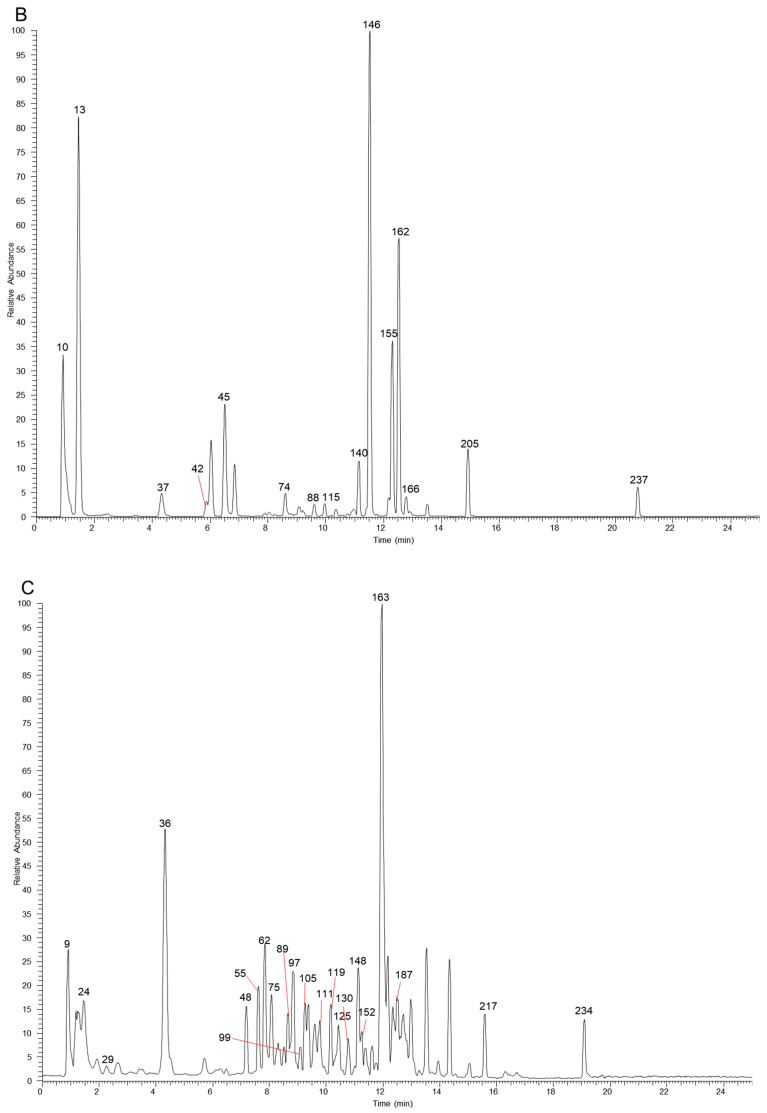

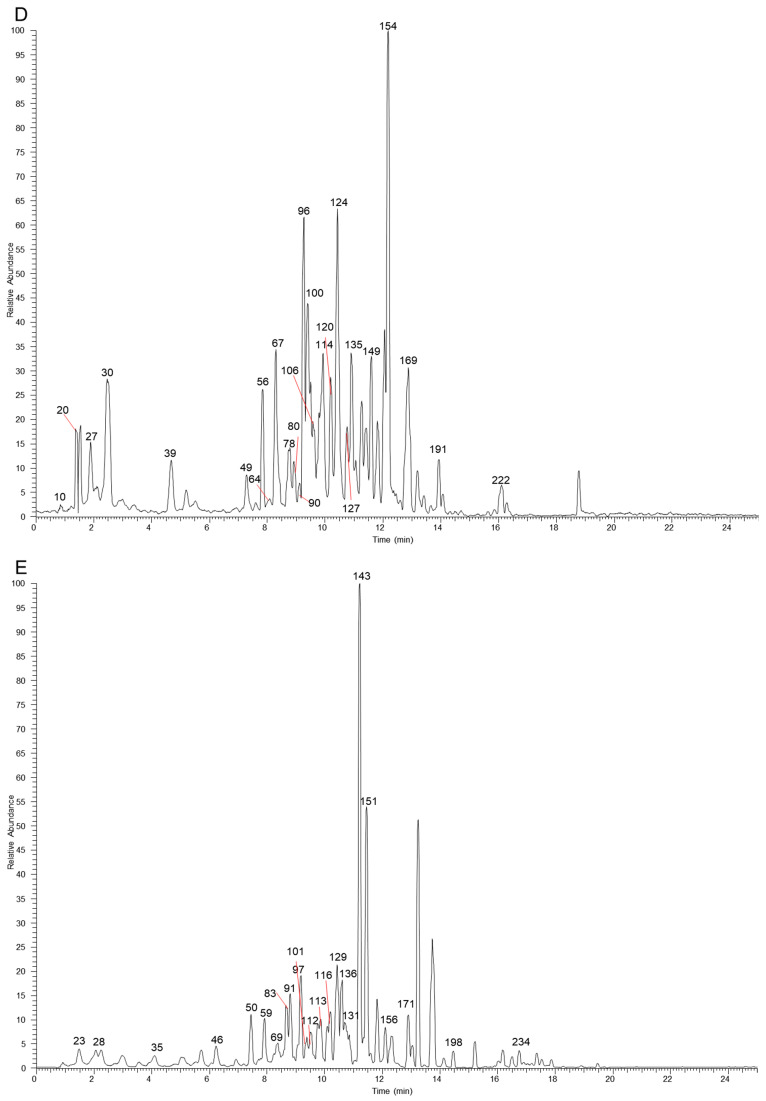
The high-resolution extracted ion chromatograms (EIC) in 10 ppm for multiple compounds in Erdun-Uril in negative mode. (**A**) *m/z* 173.0455, 355.0306, 153.0193, 183.0298, 197.0455, 515.1194, 283.0611, 267.0662, 503.3378, 329.103, 279.2329, 191.0561, 255.0662, 323.1288, 179.0349, 463.0881; (**B**) *m/z* 133.0142, 499.1668, 169.0142, 137.0244, 353.0878, 611.1617, 300.9989, 593.1511, 559.1457, 271.0611, 514.2843, 329.0666, 821.3965, 299.0924, 357.1707, 355.155; (**C**) *m/z* 331.067, 373.114, 167.0349, 403.1245, 549.1824, 387.1296, 163.04, 183.1026, 449.1089, 447.0932, 269.0455, 137.0244, 419.0983, 285.0404, 477.1038, 313.0717, 269.0819, 329.2333, 239.0713, 343.0823, 325.1445, 227.2016, 633.0733, 463.0881; (**D**) *m/z* 125.0244, 369.0463, 391.1245, 405.1402, 785.0842, 225.0768, 447.09328, 287.0561, 521.1664, 431.0983, 447.0932, 565.1926, 491.0831, 315.0146, 491.1194, 253.0506, 301.0353, 263.1288, 225.1132, 329.0666, 299.0561, 651.2658, 337.1445, 277.2173; (**E**) *m/z* 483.078, 315.0721, 285.0615, 291.0146, 595.1668, 635.0889, 151.04, 317.103, 623.1617, 599.0678, 565.1562, 167.0349, 301.0717, 287.0561, 285.0768, 345.0979, 573.2341, 271.0975, 283.0611, 335.0924, 469.3323, 315.051, 609.1461, 193.0506.

The table lists all the compounds detected in Erdun-Uril by UHPLC-Q-Exactive Orbitrap MS (Table 1 and Table S1). As the result, a total of 237 compounds (225 first reported), including 76 flavonoids, 68 phenolic compounds, 19 alkaloids, 7 amino acids, etc., were identified based on the diagnostic fragment ions, retention time, and MS^2^ database (mzVault, mzCloud, and literature). Figure 2 and Figure 3 show high-resolution ion chromatograms extracted from the Erdun-Uril extract in positive and negative ion modes, respectively.

#### 2.3.1. Identification of Flavonoids of Erdun-Uril

Flavonoids are a large class of secondary metabolites widely distributed in plants [24]. It is well known that the common structural features of most flavonoids are based on diphenylpropane A (C6-C3-C6) as the basic skeleton, where the two aromatic rings A and B are linked by a three-carbon bond [25]. Based on a comparison with fragment ion details in the literature and reference standards, we detected a total of 76 flavonoids in this study (compounds **45**, **50**, **64**–**65**, **67**, **77**, **79**–**81**, **83**–**92**, **94**–**95**, **99**–**100**, **104**, **107**, **109**, **111**, **113**, **116**, **118**–**119**, **121**–**126**, **128**–**131**, **139**–**140**, **142**–**144**, **146**–**147**, **149**–**150**, **153**–**155**, **162**, **165**–**166**, **172**–**173**, **176**–**177**, **179**, **183**, **187**, **190**, **193**, **195**, **197**–**198**, **200**–**201**). According to the connection position of the B ring of flavonoids (C-2 or C-3 position), the degree of oxidation of the central 3-carbon chain and whether the 3-carbon chain is linked, etc., the above flavonoid compounds are mainly divided into five categories: flavonoid glycoside, flavonols, dihydroflavonoids, isoflavones, and chalcones.

Compounds **64**, **77**, **79**, **80**, **86**, **87**, **84**, **121**, **124**, **131**, **139**, **140**, **150**, and **201** were identified as orientin, rutin, isoquercitrin, vitexin, hyperoside, liquiritin, isoliquiritin, kaempferol, quercetin, isorhamnetin, genistein, naringenin, liquiritigenin, and glabridin, respectively, by comparing the retention times and MS^2^ data with the reference standards.

Compounds **85** and **174** possessed the same quasi-molecular ion and characteristic fragment ions as isoliquiritigenin. Thus, they were characterized as being isoliquiritigenin isomers. Likewise, compounds **126**, **90**, **109**, **144**, and **99** were deemed to be the isomers of quercetin, orientin, liquiritin, isorhamnetin, and genistein, respectively.

Compounds **111** and **147** were found at 9.82 and 11.59 min and yielded the same quasi-molecular ion [M + H]^+^ at *m/z* 301.0706 (−1.36 ppm, C_16_H_12_O_6_); their main fragment ions [M + H]^+^ were at *m/z* 257.0436, attributed to the neutral loss of the CO_2_ (44 Da), and at *m/z* 213.0537, attributed to the neutral loss of two CO_2_ (88 Da). Combined with the literature report [12,26], they were tentatively characterized as tectorigenin and its isomer. Similarly, compounds **173** and **188**, **167** and **183**, **184** and **196**, and **84** and **113** were characterized as glycyrrhiza isoflavanone [27], glycycoumarin [28], gancaonin I, isoliquiritin apioside [28], and their isomers.

Compound **100** yielded deprotonated molecular ions [M − H]^−^ at *m/z* 491.0831 (−0.46 ppm, C_22_H_20_O_13_), and the main fragment ions [M − H]^−^ were at *m/z* 345.0809, attributed to the neutral loss of the rhamnose moiety (146 Da), and at *m/z* 300.0272 and 255.0295, were attributed to the loss of one and two COOH groups, respectively. Compound **100** had a better correspondence in both positive and negative ion modes; based on its retention time, fragmentation ions, and combined with the previous studies [29], it was confirmed as isorhamnetin 3-glucuronide. Additionally, compounds **119**, **122**, **124**, **150**, and **200** all had better correspondence in both positive and negative ion modes. According to a previous study [30,31,32], reference standards, and DFIs, they were tentatively characterized as luteolin, liquiritigenin, quercetin, isoliquiritigenin, and glabridin. Compounds **83** and **95** produced deprotonated molecular ions [M + H]^+^ at *m/z* 479.0820 (−1.43 ppm, C_21_H_18_O_13_) and 462.0792 (−0.94 ppm, C_21_H_17_O_12_). Their main fragment ions [M + H]^+^ were at *m/z* 272.0727 and 255.0797, attributed to the loss of the glucuronide, and *m/z* 113.0233 (C_5_H_5_O_3_) was their common fragment. Then, they were tentatively characterized as quercetin 3-*O*-beta-d-glucuronide and luteolin-7-*O*-beta-d-glucuronide.

Compounds **83**, **87,** and **142** yielded quasi-molecular ions [M − H]^−^ at *m/z* 317.1030 (−1.27 ppm, C_17_H_18_O_6_), 449.1089 (−0.66 ppm, C_21_H_22_O_11_), and 345.0979 (−0.74 ppm, C_18_H_18_O_7_), which initially generated 151.0310 (C_7_H_6_O_2_) and 178.9976 (C_8_H_4_O_5_) by retro Diels–Alder (RDA) rearrangement. According to previous reports [33,34,35] and DFIs, they were tentatively characterized as 3′-hydroxy-8-methoxyvestitol, carthamidin-5-glucoside, and 5,7-dihydroxy-2′,3′,4′-trimethoxyisoflavanone.

Compounds **116**, **120**, **129**, **155**, **162**, **165,** and **197** yielded deprotonated molecular ions [M − H]^−^ at *m/z* 301.0717 (−0.57 ppm, C_16_H_14_O_6_), 253.0506 (−1.63 ppm, C_15_H_10_O_4_), 285.0768 (−0.66 ppm, C_16_H_14_O_5_), 271.0975 (−1.63 ppm, C_16_H_16_O_4_), 239.0713 (−2.29 ppm, C_15_H_12_O_3_), 299.0924 (−1.13 ppm, C_17_H_16_O_5_), and 335.0924 (−0.08 ppm, C_20_H_16_O_5_), which initially generated 135.0079 (C_7_H_3_O_3_) by retro Diels–Alder (RDA) rearrangement. Thus, they were assigned as homoeriodictyol [36], soyside, sakuranetin, vestitol, 2′,4′-dihydroxychalcone, methylnissolin, and glabrone by searching in the databases such as the chemical structure database (ChemSpider) and MS^2^ database (mzVault and mzCloud) and DFIs.

#### 2.3.2. Identification of Phenolic Acids of Erdun-Uril

A total of 68 individual phenolic acids constituents were putatively identified in Erdun-Uril using UHPLC-Q-Exactive Orbitrap MS. Seven compounds, such as shikimic acid (**6**), chlorogenic acid (**42**), caffeic acid (**44**), gallic acid (**21**), ellagic acid (**74**), quinic acid (3), and ferulic acid (112) were identified as having a pseudomolecular ion [M − H]^−^ at *m/z* 173.0455 (−1.43 ppm, C_7_H_10_O_5_), 353.0878 (−0.19 ppm, C_16_H_18_O_9_), 179.0349 (−5.49 ppm, C_9_H_8_O_4_), 169.0142 (1.84 ppm, C_7_H_6_O_5_), 300.9989 (−1.30 ppm, C_14_H_6_O_8_), 191.0561 (−4.51 ppm, C_7_H_12_O_6_), and 193.0506 (−4.47 ppm, C_10_H_10_O_4_), respectively. These were unambiguously identified by comparing their accurate mass information and chromatography retention times with reference standards in negative ionization mode. Compounds **62**, **70**, and **71** were detected at 8.02, 8.44, and 8.50 min, respectively, and they possessed the same quasi-molecular ion [M − H]^−^ at *m/z* 151.0400 and the main characteristic fragment ions [M − H]^−^ at *m/z* 133.0153 (C_8_H_6_O_2_) and 107.0122 (C_7_H_8_O). In addition, compound **61** was found at 7.95 min, shared the same formula and similar fragment as compound **62**, etc., and was observed at positive scan mode. Hence, they were identified as being 4-Hydroxyphenylacetic acid isomers. Compounds **233** and **236** were eluted at 15.26 and 15.36 min with the same deprotonated ions [M + H]^+^ at *m/z* 165.0910, which initially generated 137.0593 (C_9_H_12_O) and 119.0356 (C_9_H_10_) by neutral loss CO and H_2_O, respectively. Thus, they were identified as eugenol isomers. Peaks 98 and 106 were detected at 9.38 and 9.61 min, possessed the same quasi-molecular ions [M − H]^−^ at *m/z* 315.0146, and the typical daughter ions at *m/z* 300.9984, obtained by the loss of the CH_3_ group, which further gave rise to the product ions at *m/z* 271.0463 and 227.0486 by sequential loss of a H_2_O group. In addition, *m/z* 300.9984 could be considered as a fragment of ellagic acid. Thus, compounds **98** and **106** were identified as derivatives of ellagic acid, and they were tentatively characterized as 3-O-methylellagic acid isomers. Likewise, compound **68** produced the main fragment at *m/z* 169.0131 and exhibited a similar cleavage pathway as gallic acid. Thus, it can be preliminary characterized as syringic acid. Compounds **12**, **37**, **40**, and **46** were eluted at 1.15, 4.32, 5.36, and 6.91 min with deprotonated ions [M − H]^−^ at *m/z* 355.0306 (C_14_H_12_O_11_), 137.0244 (C_7_H_6_O_3_), 183.0298 (C_8_H_8_O_5_), and 291.0146 (C_13_H_8_O_8_). According to the previous report [37,38] and DFIs, they were tentatively characterized as chebulagic acid, p-hydroxybenzoic acid, methyl gallate, and brevifolincarboxylic acid, respectively.

#### 2.3.3. Identification of Amino Acids of Erdun-Uril

Compounds **1**, **2**, **5**, **8**, **19**, and **26** were observed at 0.85, 0.87, 0.91, 0.93, 1.34, and 2.12 min, respectively, and produced deprotonated ions [M + H]^+^ at *m/z* 118.0862 (C_5_H_11_NO_2_), 116.0706 (C_5_H_9_NO_2_), 182.0811 (C_9_H_11_NO_3_), 130.0498 (C_5_H_7_NO_3_), 132.1019 (C_6_H_13_NO_2_), and 166.0862 (C_9_H_11_NO_2_). Comparing the MS^2^ fragment ions with data from the bibliography [39,40,41,42], compounds **1**, **2**, **5**, **8**, **19**, and **26** were tentatively identified as valine, proline, tyrosine, L-Pyroglutamic acid, isoleucine, and phenylalanine, respectively.

## 3. Materials and Methods

### 3.1. Chemicals and Reagents

Thermo Fisher Scientific Co., Ltd. (Fair Lawn, NJ, USA) manufactured the Dionex UltiMate 3000 Ultra Performance Liquid Chromatograph and the Q-Exactive-Orbitrap-MS. Guangzhou Watsons Food & Beverage Co., Ltd. (Guangzhou, China) provided the purified water. Kunshan Ultrasonic Instrument Co., Ltd. (Kunshan, China) manufactured the KQ-300DE sonication instrument. Aladdin Industrial Corporation (Shanghai, China) provided the other analytical grade solvents used in the experiment. Chengdu Plant Standard Pure Biotechnology Co., Ltd. (Chengdu, China) provided the chemical reference standards for chlorogenic acid, orientin, isoquercitrin, vitexin, kaempferol, isorhamnetin, caffeic acid, glabridin, and naringenin. Cheng Du Herbpurify Co., Ltd. (Chengdu, China) provided the reference standards for gallic acid and corilagin. Chengdu Aifa Biotechnology Co., Ltd., located in Chengdu, China, supplied ellagic acid, rutin, isoliquiritin, quercetin, genistein, and liquiritigenin. We acquired quinic acid, hyperoside, liquiritin, and ferulic acid from various sources, including Shanghai Yuanye Bio-Technology Co., Ltd. (Shanghai, China), the National Institutes for Food and Drug Control, Sigma Aldrich (Shanghai) Trading Co., Ltd. (Shanghai, China), and Shandong West Asia Chemical Technology Co., Ltd. (Linyi, China), respectively. All reference standards had purities exceeding 98% as determined by HPLC-UV analysis.

### 3.2. Preparation of Specimen and Standard Solution

Erdun-Uril (211102) was acquired from Inner Mongolia Kulun Mongolian Pharmaceutical Co., Ltd. (Tongliao, China)

Prior to sample preparation, we powdered the Erdun-Uril substance and then passed it through a No. 40 mesh sieve. We treated the Erdun-Uril powder (2 g) with sonication (300 W, 40 kHz) in 10 mL of 70% (*v/v*) methanol for 1 h at room temperature (16–24 °C). The final product was then filtered and dried using rotary evaporation and nitrogen blowing. We then dissolved the solution again using methanol and centrifuged it at a speed of 12,000 rpm for 15 min to obtain the supernatant. For analysis using UHPLC-Q-Exactive Orbitrap MS, a total of 2 μL of material was prepared.

Standard solutions were prepared in methanol at a concentration of 1 mg/mL. These reference standard solutions were further diluted to obtain working solutions and then were stored at 4℃ before analysis.

### 3.3. Instruments and Conditions

In order to obtain a better chromatographic peak shape and separation resolution, various factors were set in the detection and identification process, including a column (Agilent Eclipse Plus C8 2.1 × 100 mm, 1.8 μm), column temperature (40 °C), and the mobile phase gradient.

Each LC-MS analysis was exercised on a Q-Exactive Focus Orbitrap MS connected to a Thermo Scientific Dionex Ultimate 3000 RS (Thermo Fisher Scientific Co., Ltd., Waltham, MA, USA) through an ESI source. Chromatographic separation was performed at 40 °C using an Agilent Eclipse Plus C8 (2.1 × 100 mm, 1.8 μm). The mobile phase was composed of 0.1% formic acid (A) and acetonitrile (B), and the flow rate was 0.3 mL/min. The following gradient was used: 0–2 min, 95–92%A; 2–5 min, 92–90%A; 5–12 min, 90–45%A; 12–18 min, 45–15%A; 18–23 min, 15–10%A; 23–25 min, 10–5% A; 25–30 min, 5–95%A.

All data were obtained in alternating positive and negative ion scanning mode using the following tuning method. In terms of the mass spectrometry conditions, the spray voltage was 3.5 kV in positive ion mode and 3.0 kV in negative ion mode. The spray voltage was 3.0 kV; the sheath gas and auxiliary gas pressure were 30 arb and 10 arb; the capillary temperature was 320 °C; the S-lens RF level was 50. In the mass range of *m/z* 100–1500, a high-resolution mass spectrum was obtained at a resolution of 70,000, which was detected by the Orbitrap analyzer. MS^2^ data at a resolution of 17,500 were obtained by data-dependent MS^2^ scanning or parallel reaction monitoring (PRM) mode. Nitrogen (purity ≥ 99.999%) served as the collision gas, which generated the fragment ions, and the energy level was set as a normalized collision energy of 30%.

### 3.4. Data Processing and Analysis

Xcalibur software version 4.2 (Thermo Fisher Scientific, San Jose, CA, USA) was used to obtain all high-resolution data, including the full-scan MS and MS^2^ data. Peaks detected with intensities over 10,000 were selected for identification. The chemical formulas for all parent and fragment ions of the selected peaks were calculated from the accurate mass using a formula predictor by setting the parameters as follows: C [0–90], H [0–90], O [0–90], and N [0–10]. The mass tolerance of MS and MS^2^ was within 10 ppm.

## 4. Conclusions

In this study, an efficient strategy based on UHPLC-Q-Exactive Orbitrap MS was established to detect chemical components in Erdun-Uril. A total of 237 constituents were identified, of which 225 were first reported in Erdun-Uril, including 76 flavonoids, 68 phenolic acids, 19 alkaloids, 7 amino acids, etc. These were detected and identified based on their chromatographic retention, MS and MS^2^, and bibliography data. These results are very useful for understanding the bioactive compounds of Erdun-Uril and their utilization. Overall, the results lay the foundation for in-depth research on the pharmacodynamic material basis of Erdun-Uril.

## Figures and Tables

**Table 1:** Identification results of the chemical composition of Erdun-Uril.

No	*t_R_*/min	Formula (M)	Identification	No	*t_R_*/min	Formula (M)	Identification
1	0.85 ^#^	C_5_H_11_NO_2_	Valine	121	10.30 *^#^	C_15_H_10_O_6_	Kaempferol
2	0.87 ^#^	C_5_H_9_NO_2_	Proline	122	10.32 *^#^	C_15_H_12_O_4_	Liquiritigenin
3	0.88 *	C_7_H_12_O_6_	Quinic acid	122	10.33 *^#^	C_15_H_12_O_4_	Liquiritigenin
4	0.89 ^#^	C_5_H_5_N_5_O	2-Hydroxyadenine	123	10.39 ^#^	C_15_H_12_O_6_	Eriodictyol
5	0.91 ^#^	C_9_H_11_NO_3_	Tyrosine	124	10.43 *^#^	C_15_H_10_O_7_	Quercetin
6	0.91 ^#^	C_7_H_10_O_5_	Shikimic acid	125	10.46 ^#^	C_22_H_22_O_12_	Isorhamnetin-3-*O*-glucoside
7	0.92 ^#^	C_6_H_5_NO_2_	Nicotinic acid	126	10.47 ^#^	C_15_H_10_O_7_	Quercetin isomer
8	0.93 ^#^	C_5_H_7_NO_3_	L-Pyroglutamic acid	124	10.47 *^#^	C_15_H_10_O_7_	Quercetin
9	0.94 ^#^	C_13_H_16_O_10_	Glucogallin	127	10.54 ^#^	C_15_H_20_O_4_	Abscisic acid
10	0.99 ^#^	C_4_H_6_O_5_	Malic acid	128	10.59 ^#^	C_16_H_12_O_5_	Genkwanin
11	1.08 ^#^	C_5_H_5_N_5_	Adenine	129	10.68 ^#^	C_16_H_14_O_5_	Sakuranetin
12	1.15 ^#^	C_14_H_12_O_11_	Chebulagic acid	130	10.79 ^#^	C_17_H_14_O_6_	Pectolinarigenin
13	1.17 ^#^	C_19_H_32_O_15_	3-{[6-*O*-(d-galactopyranosyl)-β-d-galactopyranosyl] oxy}-1,2-propan-ediyldiace-tate	131	10.81 *^#^	C_16_H_12_O_7_	Isorhamnetin
14	1.18 ^#^	C_9_H_8_O_3_	P-Hydroxycinnamic acid	132	10.85 ^#^	C_15_H_20_O_3_	Santamarine
15	1.19 ^#^	C_5_H_5_N_5_O	Guanine	133	10.90 ^#^	C_17_H_17_O_5_Cl	8-Chloro-2-(2-phenylethyl)-5,6,7-trihydroxy-5,6,7,8-tetrahydrochromone
16	1.27 ^#^	C_10_H_13_N_5_O_4_	Adenosine	134	10.90 ^#^	C_27_H_36_O_11_	6′-*O*-trans-sinapoyl jasminoside A
17	1.2 8^#^	C_4_H_6_O_4_	Succinic Acid	135	10.93 ^#^	C_12_H_18_O_4_	Senkyunolide
18	1.29 ^#^	C_13_H_16_O_10_	1-Galloyl-beta-glucose	136	10.96 ^#^	C_23_H_22_O_13_	3,4,3′-trimethylated ellagic acid-4′-*O*-beta-d-glucoside
19	1.34 ^#^	C_6_H_13_NO_2_	Isoleucine	137	10.98 ^#^	C_42_H_62_O_18_	Glycyrrhizin G2
20	1.37 ^#^	C_6_H_6_O_3_	Pyrogallic acid	138	11.08 ^#^	C_15_H_22_O	Germacrone
21	1.45 *	C_7_H_6_O_5_	Gallic acid	139	11.13 *^#^	C_15_H_10_O_5_	Genistein
22	1.47 ^#^	C_13_H_16_O_10_	Gallic acid 6-*O*-β-d-glucopyranoside	140	11.14 *^#^	C_15_H_12_O_5_	Naringenin
23	1.50 ^#^	C_20_H_20_O_14_	1,6-di-*O*-galloyl-β-d-glucose	141	11.18 ^#^	C_9_H_6_O_4_	Esculetin
24	1.92 ^#^	C_16_H_22_O_10_	Gardoside	119	11.24 ^#^	C_15_H_10_O_6_	Luteolin
25	2.07 ^#^	C_20_H_20_O_14_	3,6-di-*O*-galacyl-d-glucose	142	11.33 ^#^	C_18_H_18_O_7_	5,7-dihydroxy-2′,3′,4′-trimethoxy isoflavanone
26	2.12 ^#^	C_9_H_11_NO_2_	Phenylalanine	143	11.35 ^#^	C_16_H_12_O_7_	Isorhamnetin isomer
27	2.13 ^#^	C_15_H_14_O_11_	Methyl-13-chebulaic acid	144	11.46 ^#^	C_16_H_12_O_5_	Wogonin
28	2.18 ^#^	C_13_H_16_O_9_	2-hydroxy-3-[(2S,3R,4S,5S,6R)-3,4,5-trihydroxy-6-(hydroxymethyl)oxan-2-yl]oxybenzoic acid	145	11.52 ^#^	C_26_H_45_NO_7_S	Cholic acid
29	2.25 ^#^	C_8_H_8_O_4_	Vanillic acid	146	11.59 ^#^	C_16_H_12_O_6_	Tectorigenin isomer
30	2.49 ^#^	C_16_H_24_O_11_	Shanzhiside	147	11.61 ^#^	C_16_H_14_O_4_	2′-methoxyisoliquiritigenin
31	2.65 ^#^	C_16_H_22_O_10_	Geniposidic acid	148	11.77 ^#^	C_20_H_22_O_4_	Aurantio-obtusin isomer
32	2.66 ^#^	C_7_H_6_O_4_	Protocatechuic acid	149	11.78 *	C_15_H_12_O_4_	Isoliquiritigenin
33	3.39 ^#^	C_6_H_6_O_3_	Maltol	150	11.78 *	C_15_H_12_O_4_	Isoliquiritigenin
34	3.39 ^#^	C_6_H_6_O_3_	5-Hydroxymethylfurfural	150	11.84 ^#^	C_30_H_38_O_11_	Isotoosendanin
35	4.09 ^#^	C_12_H_14_O_8_	Uralenneoside	151	11.95 ^#^	C_18_H_34_O_5_	9(S),10(S),11(R)-trihydroxy-12(Z)-octadecenoic acid
36	4.26 ^#^	C_17_H_24_O_11_	Scandioside methyl ester	152	11.98 ^#^	C_16_H_19_NO_2_	Medifoxamine
37	4.32 ^#^	C_7_H_6_O_3_	p-hydroxybenzoic acid	153	12.05 ^#^	C_16_H_12_O_6_	Hydroxygenkwanin
38	4.65 ^#^	C_6_H_11_NO	N-Formylpiperidine	154	12.10 ^#^	C_16_H_12_O_4_	Formononetin
39	4.69 ^#^	C_17_H_26_O_11_	Shanzhiside methylester	155	12.11^#^	C_16_H_16_O_4_	Vestitol
40	5.36 ^#^	C_8_H_8_O_5_	Methyl gallate	156	12.11 ^#^	C_30_H_38_O_11_	Toosendanin
41	5.88 ^#^	C_9_H_6_O_3_	7-Hydroxycoumarin	157	12.18 ^#^	C_17_H_14_O_7_	Aurantio-obtusin
42	5.88 *	C_16_H_18_O_9_	Chlorogenic acid	158	12.18 ^#^	C_17_H_14_O_7_	Cirsiliol
42	5.88 *	C_16_H_18_O_9_	Chlorogenic acid	159	12.26 ^#^	C_14_H_17_NO_3_	Fagaramide
43	5.99 ^#^	C_16_H_26_O_8_	Jasminoside B/D/G	160	12.27 ^#^	C_30_H_48_O_6_	Arjugenin
44	6.29 *	C_9_H_8_O_4_	Caffeic acid	161	12.30 ^#^	C_42_H_62_O_16_	Glycyrrhizic acid
45	6.51 ^#^	C_27_H_32_O_16_	Safflomin A	162	12.30 ^#^	C_42_H_62_O_16_	Glycyrrhizic acid
46	6.91 ^#^	C_13_H_8_O_8_	Brevifolincarboxylic acid	162	12.33 ^#^	C_15_H_12_O_3_	2′,4′-dihydroxychalcone
47	7.18 ^#^	C_10_H_8_O_3_	4-Methylumbelliferone	163	12.35 ^#^	C_18_H_18_O_6_	3′-*O*-methylviolanone
48	7.19 ^#^	C_23_H_34_O_15_	Genipin 1-*O*-beta-d-Gentiobioside	164	12.52 ^#^	C_9_H_10_O	Cinnamic alcohol
48	7.19 ^#^	C_23_H_34_O_15_	Genipin 1-*O*-beta-d-Gentiobioside	165	12.52 ^#^	C_17_H_16_O_5_	Methylnissolin
49	7.29 ^#^	C_34_H_26_O_22_	Tellimagradin I	166	12.62 ^#^	C_21_H_20_O_6_	Glycycoumarin
50	7.41 ^#^	C_27_H_32_O_15_	Isobutrin	167	12.65 ^#^	C_16_H_17_NO_3_	Piperyline
51	7.57 ^#^	C_15_H_14_O_6_	Epicatechin	168	12.77	C_32_H_44_O_14_	Crocin III
52	7.61 ^#^	C_11_H_12_O_5_	Sinapic acid	169	12.83 ^#^	C_17_H_19_NO_5_	Piperodione
53	7.61 *	C_27_H_22_O_18_	Corilagin	170	12.91 ^#^	C_16_H_12_O_5_	Physcione
54	7.81 ^#^	C_9_H_10_O_3_	Paeonol	171	12.97 ^#^	C_17_H_14_O_3_	Benzarone
55	7.82 ^#^	C_17_H_24_O_10_	Geniposide	172	13.09 ^#^	C_20_H_18_O_6_	Glycyrrhiza isoflavanone
56	7.8 3^#^	C_11_H_14_O_5_	Genipin	173	13.10 ^#^	C_15_H_12_O_4_	Isoliquiritigenin isomers
57	7.84 ^#^	C_8_H_7_N	Indole	174	13.17 ^#^	C_16_H_19_NO_3_	Piperlonguminine
58	7.88 ^#^	C_11_H_12_O_4_	Ethyl caffeate	175	13.18 ^#^	C_24_H_38_O_4_	12-ketolcholic acid
59	7.90 ^#^	C_27_H_24_O_18_	1,2,6-triple-*O*-galacyl-β -d-glucose	176	13.24 ^#^	C_16_H_12_O_5_	Pea chalcone B
60	7.95 ^#^	C_15_H_20_O_3_	Parthenolide	177	13.24 ^#^	C_16_H_12_O_5_	Melanettin
61	7.95 ^#^	C_8_H_8_O_3_	4-Hydroxyphenylacetic acid isomer	178	13.28 ^#^	C_15_H_22_	α-curcumene
62	8.02 ^#^	C_8_H_8_O_3_	4-Hydroxyphenylacetic acid isomer	179	13.28 ^#^	C_21_H_20_O_5_	Gancaonin M
63	8.20 ^#^	C_15_H_20_O_3_	Pterosin A	180	13.29 ^#^	C_20_H_22_O_4_	Machilin A
64	8.28 *^#^	C_21_H_20_O_11_	Orientin	181	13.29 ^#^	C_20_H_24_O_5_	Fragransin A2
65	8.29 ^#^	C_26_H_28_O_14_	Schaftoside	182	13.37 ^#^	C_21_H_20_O_6_	Glycycoumarin isomer
66	8.34 ^#^	C_10_H_16_O_2_	Chrysanthemic acid	183	13.45 ^#^	C_21_H_22_O_5_	Gancaonin I
67	8.35 ^#^	C_15_H_12_O_6_	Dihydrofisetin	184	13.52 ^#^	C_19_H_18_O_4_	Tanshinaldehyde
68	8.40 ^#^	C_9_H_10_O_5_	Syringic acid	185	13.54 ^#^	C_17_H_21_NO_3_	Piperanine
69	8.42 ^#^	C_9_H_8_O_3_	p-Coumaric acid	186	13.55 ^#^	C_18_H_16_O_7_	Obtusin
70	8.44 ^#^	C_8_H_8_O_3_	4-Hydroxyphenylacetic acid isomer	187	13.60 ^#^	C_20_H_18_O_6_	Glycyrrhiza isoflavanone isomer
71	8.50 ^#^	C_8_H_8_O_3_	4-Hydroxyphenylacetic acid isomer	188	13.87	C_11_H_12_O_3_	Myristicin
72	8.52 ^#^	C_9_H_10_O_4_	4-Hydroxy-3,5-dimethoxybenzaldehyde	189	13.94 ^#^	C_30_H_46_O_3_	Wilforlide A
73	8.52 ^#^	C_9_H_10_O_4_	Methyl oxalate	190	13.94 ^#^	C_21_H_22_O_4_	Licochalcone A
74	8.62 *^#^	C_14_H_6_O_8_	Ellagic acid	191	14.06 ^#^	C_10_H_10_O_2_	Safrol
75	8.65 ^#^	C_10_H_16_O_3_	Jasminodiol	192	14.06 ^#^	C_30_H_20_O	Tetrahydropyranthron
76	8.69 ^#^	C_10_H_8_O_4_	Scopoletin	193	14.08 ^#^	C_21_H_18_O_6_	Glycyrol
77	8.69 *^#^	C_27_H_30_O_16_	Rutin	194	14.17 ^#^	C_17_H_14_O_2_	2- (2-phenylethyl) chromone
78	8.70 ^#^	C_25_H_30_O_12_	2′(4′-hydroxycinnamoyl)–Polygonatum japonicum glycoside	195	14.20 ^#^	C_21_H_22_O_5_	Gancaonin I isomer
79	8.75 *^#^	C_21_H_20_O_12_	Isoquercitrin	196	14.43 ^#^	C_18_H_16_O_2_	Cinnamyl cinnamate
80	8.77 *^#^	C_21_H_20_O_10_	Vitexin	197	14.46 ^#^	C_20_H_16_O_5_	Glabrone
81	8.80 ^#^	C_21_H_18_O_13_	Quercetin3-*O*-β-d-Glucuronide	198	14.47 ^#^	C_18_H_16_O_3_	Ipriflavone
82	8.82 ^#^	C_17_H_18_O_6_	Agarotetrol	199	14.54 ^#^	C_19_H_21_NO_3_	Piperine
83	8.82 ^#^	C_17_H_18_O_6_	3′-hydroxy-8-methoxyvestitol	200	14.62 *^#^	C_20_H_20_O_4_	Glabridin
84	8.84 ^#^	C_26_H_30_O_13_	Isoliquiritin	200	14.62 *^#^	C_20_H_20_O_4_	Glabridin
85	8.85 ^#^	C_15_H_12_O_4_	Isoliquiritigenin isomers	201	14.69 ^#^	C_19_H_23_NO_3_	Piperdardine
86	8.85 *^#^	C_21_H_20_O_12_	Hyperoside	202	14.88	C_15_H_20_O_2_	Isoalantolactone
87	8.86 *^#^	C_21_H_22_O_9_	Liquiritin	203	14.88	C_15_H_20_O_2_	Costunolide
88	9.08	C_27_H_30_O_15_	Kaempferol-3-*O*-rutinoside	204	14.92 ^#^	C_21_H_26_O_5_	Malabaricone C
88	9.08	C_27_H_30_O_15_	Kaempferol-3-*O*-rutinoside	205	14.97 ^#^	C_10_H_12_O_2_	P-Hydroxyphenyl butanone
89	9.09 ^#^	C_21_H_22_O_11_	Carthamidin-5-glucoside	206	14.97 ^#^	C_11_H_14_O_3_	Methoxyeugenol
90	9.11 ^#^	C_21_H_20_O_11_	Orientin isomer	207	15.14 ^#^	C_15_H_18_O_2_	Lindenenol
91	9.18 ^#^	C_28_H_32_O_16_	Narcissoside	208	15.14 ^#^	C_15_H_18_O_2_	Dehydro-α-curcumene
92	9.23 ^#^	C_21_H_17_O_12_	Luteolin-7-*O*-beta-d-glucuronide	209	15.14 ^#^	C_20_H_25_NO_3_	Piperlongumine A
93	9.24 ^#^	C_25_H_24_O_12_	Isochlorogenic acid B	210	15.26 ^#^	C_10_H_12_O_2_	Eugenol isomer
94	9.26 ^#^	C_21_H_20_O_11_	Cynaroside	211	15.30 ^#^	C_17_H_24_O_4_	9-Acetoxyfukinanolide
95	9.27 ^#^	C_20_H_18_O_7_	Uralenol	212	15.35	C_14_H_25_NO	Pellitorine
96	9.31 ^#^	C_27_H_34_O_13_	11-(6-*O*-trans-sinapoyl-glucopyranosyl) gardendiol	213	15.36 ^#^	C_10_H_12_O_2_	Eugenol isomer
97	9.36 ^#^	C_27_H_20_O_16_	4-*O*-(4′-*O*-galloyl-rhamnosyl) ellagic acid	214	15.47 ^#^	C_20_H_27_NO_3_	Pipercallosine
98	9.38 ^#^	C_15_H_8_O_8_	3-*O*-methylellagic acid isomer	215	15.55 ^#^	C_15_H_20_O_2_	Atractylenolide
99	9.38 ^#^	C_15_H_10_O_5_	Genistein isomer	216	15.58	C_20_H_22_O_4_	Dehydrodiisoeugenol
100	9.39 ^#^	C_22_H_20_O_13_	Isorhamnetin 3-glucuronide	217	15.71	C_20_H_22_O_4_	Dehydrodiisoeugenol
100	9.40 ^#^	C_22_H_20_O_13_	Isorhamnetin 3-glucuronide	217	15.71 ^#^	C_21_H_25_NO_3_	Piptigrine
101	9.43 ^#^	C_26_H_30_O_14_	Cassiaside B	218	15.97 ^#^	C_15_H_24_O	Spathulenol
102	9.46 ^#^	C_10_H_10_O_3_	6,7-dihydroxyindan-4-carbaldehyde	219	15.98 ^#^	C_15_H_25_NO	Neopellitorine B
103	9.48 ^#^	C_25_H_24_O_12_	3,5-di-caffeoylquinic acid	220	16.08 ^#^	C_21_H_27_NO_3_	Pipernonaline
104	9.57 ^#^	C_9_H_6_O_2_	Coumarin	221	16.13	C_18_H_30_O_2_	α-linolenic acid
105	9.59 ^#^	C_7_H_6_O_3_	3-Hydroxybenzoic acid	222	16.18 ^#^	C_12_H_16_O_3_	Isoelemicine
106	9.61 ^#^	C_15_H_8_O_8_	3-O-methylellagic acid	223	16.18 ^#^	C_12_H_16_O_3_	α-Asarone
107	9.62 ^#^	C_15_H_10_O_5_	Emodin	224	16.27 ^#^	C_11_H_14_O_2_	Methyleugenol
108	9.76 ^#^	C_8_H_8_O_4_	Vanillic acid isomer	225	16.32 ^#^	C_18_H_34_O_2_	Oleic acid
109	9.77^#^	C_21_H_22_O_9_	Liquiritin isomer	226	16.55 ^#^	C_21_H_29_NO_3_	Piperolein B
110	9.81 ^#^	C_20_H_20_O_10_	Cassiaside	227	16.88 ^#^	C_16_H_22_O_4_	Mansonone N
111	9.8 2^#^	C_16_H_12_O_6_	Tectorigenin	228	16.98 ^#^	C_16_H_22_O_4_	Dibutyl phthalate
112	9.86 *^#^	C_10_H_10_O_4_	Ferulic acid	229	17.14 ^#^	C_16_H_29_NO	N-isobutyl1-2,4-dodecadienamide
113	9.88^#^	C_26_H_30_O_13_	Isoliquiritin apioside isomer	230	17.25 ^#^	C_18_H_22_O_4_	Nordihydroguaiaretic acid
114	9.93 ^#^	C_23_H_24_O_12_	Aurantio-obtusin-6-*O*-β-d-glucoside	231	17.36 ^#^	C_30_H_46_O_4_	18-β-glycyrrhetinic acid
115	9.96 ^#^	C_27_H_28_O_13_	4-*O*-sinapoyl-5-*O*-caffeoylquninic acid	232	17.83 ^#^	C_24_H_33_NO_3_	Guineensine
84	10.07 *^#^	C_21_H_22_O_9_	Isoliquiritin	233	19.09 ^#^	C_14_H_28_O_2_	Myristic acid
116	10.09 ^#^	C_16_H_14_O_6_	Homoeriodictyol	234	19.50 ^#^	C_23_H_35_NO	Dihydroevocarpine
117	10.16 ^#^	C_15_H_8_O_5_	Coumestrol	235	19.75 ^#^	C_18_H_32_O_2_	Linoleic acid
118	10.18 ^#^	C_22_H_22_O_9_	Ononin	236	20.79 ^#^	C_21_H_24_O_5_	Rutamarin
119	10.18 ^#^	C_15_H_10_O_6_	Luteolin	237	22.30^#^	C_22_H_41_NO	N-isobutyl-(2E,4E)-octadecadienamide
120	10.20 ^#^	C_15_H_10_O_4_	Chrysophanic acid				

* Identified by comparison with standards. ^#^ First reported in Erdun-Uril.

## Data Availability

Data will be provided upon request.

## Supplementary Materials

The following Supporting Information can be downloaded at: https://www.mdpi.com/article/10.3390/molecules29184349/s1; Table S1: Retention times and mass spectral data of Erdun-Uril.
