# Supplementary material for: A Systematic Methodology for the Identification of the Chemical Composition of the Mongolian Drug Erdun-Uril Compound Utilizing UHPLC-Q-Exactive Orbitrap Mass Spectrometry
Source: Molecules. 2024 Sep 13;29(18):4349. doi: 10.3390/molecules29184349 (PMC11434484; doi:10.3390/molecules29184349)
Supplement: Supplementary file 1 [file molecules-29-04349-s001.zip › molecules-3154508-supplementary.pdf]

Table S1. Chromatographic and mass data of the components detected in Erdun-Uril through UHPLC-Q-Exactive Orbitrap MS.

No	t_R/min	m/z		Error (ppm)	Formula (M)	MS/MS fragment		Identification
		Theoretical Mass	Experimental Mass			$[M+H]^+$	$[M-H]^-$	
1	0.85$^{\#}$	118.0862	118.0862	-0.47	$C_5H_{11}NO_2$	MS^2[118]: 118.0861(100) 59.0735(7) 58.0657(5)		Valine
2	0.87$^{\#}$	116.0706	116.0705	-0.13	$C_5H_9NO_2$	MS^2[116]: 70.0656(100) 116.0705(34)		Proline
3	0.88*	191.0561	191.0552	-4.51	$C_7H_{12}O_6$		MS^2[191]: 111.0074(100) 191.0552(13) 87.0073(51) 85.0280(37) 59.0124(2)	Quinic acid
4	0.89$^{\#}$	152.0566	152.0564	-1.82	$C_5H_5N_5O$	MS^2[152]: 152.0564(100) 153.0403(12) 135.0298(11) 110.0349(5)		2-Hydroxyadenine
5	0.91$^{\#}$	182.0811	182.0809	-1.32	$C_9H_{11}NO_3$	MS^2[182]: 136.0753(100) 123.0438(49) 165.0542(19) 147.0436(19) 119.0490(29)		Tyrosine

6	0.91#	173.0455	173.0453	-1.43	$C_7H_{10}O_5$		MS^2[173]:<br>93.0332(100)<br>73.0280(56)<br>137.0232(29)<br>111.0074(43)	Shikimic acid
7	0.92#	124.0393	124.0392	-0.60	$C_6H_5NO_2$	MS^2[124]:<br>124.0391(100)<br>96.0446(6)		Nicotinic acid
8	0.93#	130.0498	130.0497	-0.99	$C_5H_7NO_3$	MS^2[130]:<br>84.0447(100)<br>84.0810(82)<br>130.0860(43)<br>130.0495(20)<br>112.0868(12)<br>70.0656(53)		L-Pyroglutamic acid
9	0.94#	331.0670	331.0670	-0.15	$C_{13}H_{16}O_{10}$		MS^2[331]:<br>169.0131(100)<br>271.0452(6)<br>211.0241(26)<br>125.0231(29)	Glucogallin
10	0.99#	133.0142	133.0139	-2.73	$C_4H_6O_5$		MS^2[133]:<br>115.0023(100)<br>71.0124(45)<br>133.0129(34)	Malic acid
11	1.08#	136.0617	136.0616	-0.90	$C_5H_5N_5$	MS^2[136]:<br>136.0614(100)<br>136.0214(2)<br>137.0455(1)		Adenine
12	1.15#	355.0306	355.0304	-0.72	$C_{14}H_{12}O_{11}$		MS^2[355]:<br>205.0498(100)<br>111.0074(17)	Chebulagic acid

							93.0074(9)<br>161.0597(72)<br>163.0389(96)<br>193.0134(85)	
13	1.17#	499.1668	499.1666	-0.37	$C_{19}H_{32}O_{15}$		MS^2[499]:<br>93.0331(100)<br>191.0552(40)<br>173.0445(48)<br>111.0438(22)	3-{[6-O-(D-galactopyrano-syl)-β-D-galactopyranosyl] oxy}-1,2-propan-ediyldiace-tate
14	1.18#	165.0546	165.0543	-1.46	$C_9H_8O_3$	MS^2[165]:<br>123.0439(100)<br>139.0490(41)<br>147.0436(14)		P-Hydroxycinnamic acid
15	1.19#	152.0566	152.0564	-1.42	$C_5H_5N_5O$	MS^2[152]:<br>152.0563(100)<br>135.0298(10)<br>110.0349(4)		Guanine
16	1.27#	268.1040	268.1036	-1.46	$C_{10}H_{13}N_5O_4$	MS^2[268]:<br>152.0562(100)<br>136.0612(2)<br>117.0547(4)<br>71.0492(1)<br>57.0339(1)<br>150.0288(1)		Adenosine
17	1.28#	117.0193	117.0180	-2.55	$C_4H_6O_4$		MS^2[117]:<br>73.0280(100)<br>117.0180(27)<br>116.9272(10)	Succinic Acid
18	1.29#	331.0670	331.0670	0.03	$C_{13}H_{16}O_{10}$		MS^2[331]:<br>169.0132(100)<br>211.0240(48)	1-Galloyl-beta-glucose

							125.0231(32)<br>271.0456(14)	
19	1.34#	132.1019	132.1017	-1.10	$C_6H_{13}NO_2$	MS^2[132]:<br>86.0967(100)<br>132.1017(2)		Isoleucine
20	1.37#	125.0244	125.0243	-0.58	$C_6H_6O_3$		MS^2[125]:<br>125.0231(100)<br>107.0126(2)	Pyrogallic acid
21	1.45*	169.0142	169.0144	1.84	$C_7H_6O_5$		MS^2[169]:<br>125.0231(100)<br>93.0280(2)<br>81.0331(2)<br>169.0131(20)	Gallic acid
22	1.47#	331.0670	331.0667	-0.91	$C_{13}H_{16}O_{10}$		MS^2[331]:<br>169.0131(100)<br>125.0231(23)<br>211.0240(67)<br>271.0458(23)<br>125.0231(23)<br>168.0052(9)	Gallic acid 6-O-β-D-glucopyranoside
23	1.50#	483.0780	483.0779	-0.25	$C_{20}H_{20}O_{14}$		MS^2[483]:<br>169.0131(100)<br>331.0673(3)<br>313.0553(3)<br>125.0231(40)	1,6-di-O-galloyl-β-D-glucose
24	1.92#	373.1140	373.1136	-1.00	$C_{16}H_{22}O_{10}$		MS^2[373]:<br>123.0438(100)<br>149.0598(38)<br>89.0230(39)<br>71.0124(24)<br>59.0124(56)	Gardoside

							193.0492(21)	
25	2.07#	483.0780	483.0776	-0.74	$C_{20}H_{20}O_{14}$		MS^2[483]: 169.0131(100) 211.0246(6) 331.0669(4) 313.0559(5)	3,6-di-O-galacyl-D-glucose
26	2.12#	166.0862	166.0859	-1.66	$C_9H_{11}NO_2$	MS^2[166]: 120.0806(100) 166.0858(1) 107.0492(2) 121.0758(1)		Phenylalanine
27	2.13#	369.0463	369.0460	-0.69	$C_{15}H_{14}O_{11}$		MS^2[369]: 205.0500(96) 193.0133(100) 187.0387(40) 161.0594(64) 137.0645(37)	Methyl-13-chebulaic acid
28	2.18#	315.0721	315.0721	-0.14	$C_{13}H_{16}O_9$		MS^2[315]: 152.0382(100) 108.0202(81) 109.0283(32) 153.0182(33)	2-hydroxy-3-[(2S,3R,4S,5S,6R)-3,4,5-trihydroxy-6-(hydroxymethyl)oxan-2-yl]oxybenzoic acid
29	2.25#	167.0349	167.0348	-0.37	$C_8H_8O_4$		MS^2[167]: 123.0438(100) 138.0387(41) 149.0239(16)	Vanillic acid
30	2.49#	391.1245	391.1244	-0.34	$C_{16}H_{24}O_{11}$		MS^2[391]: 229.0705(7) 185.0812(28) 167.0704(41)	Shanzhiside

							149.0596(55)<br>121.0645(27)<br>119.0337(32)<br>59.0124(100)<br>71.0124(57)	
31	2.65#	373.1140	373.1136	-1.00	$C_{16}H_{22}O_{10}$		MS^2[373]:<br>123.0439(100)<br>149.0596(75)<br>89.0229(23)<br>71.0122(11)<br>59.0124(26)	Geniposidic acid
32	2.66#	153.0193	153.0190	-1.40	$C_7H_6O_4$		MS^2[153]:<br>109.0281(100)<br>91.0175(1)<br>81.0331(2)<br>65.0382(1)	Protocatechuic acid
33	3.39#	127.0389	127.0388	-0.71	$C_6H_6O_3$	MS^2[127]:<br>127.0388(100)<br>109.1012(12)<br>81.0702(2)		Maltol
34	3.39#	127.0389	127.0388	-0.71	$C_6H_6O_3$	MS^2[127]:<br>127.0388(100)<br>109.1012(18)<br>81.0338(5)		5-Hydroxymethylfurfural
35	4.09#	285.0615	285.0614	-0.67	$C_{12}H_{14}O_8$		MS^2[285]:<br>153.0069(100)<br>109.0282(96)<br>108.0203(22)<br>152.0104(45)	Uralenneoside
36	4.26#	403.1245	403.1241	-1.00	$C_{17}H_{24}O_{11}$		MS^2[403]:<br>127.0387(100)	Scandioside methyl ester

							191.0339(15)<br>161.0230(18)<br>101.0230(66)<br>71.0125(27)<br>68.9968(7)<br>59.0124(48)	
37	4.32#	137.0244	137.0242	-0.81	$C_7H_6O_3$		MS^2[137]:<br>119.0281(2)<br>93.0330(2)	p-hydroxybenzoic acid
38	4.65#	114.0913	114.0914	0.61	$C_6H_{11}NO$	MS^2[114]:<br>114.0912(100)<br>69.0704(3)<br>72.0812(4)		N-Formylpiperidine
39	4.69#	405.1402	405.1399	-0.78	$C_{17}H_{26}O_{11}$		MS^2[405]:<br>197.0810(74)<br>113.0228(11)<br>59.1023(9)<br>153.0544(8)	Shanzhiside methylester
40	5.36#	183.0298	183.0289	-5.34	$C_8H_8O_5$		MS^2[183]:<br>124.0152(100)<br>139.0024(47)<br>111.0074(60)<br>95.0125(12)	Methyl gallate
41	5.88#	163.0389	163.0386	-1.78	$C_9H_6O_3$	MS^2[163]:<br>135.0438(100)<br>163.0386(80)<br>145.0281(46)<br>117.0334(37)<br>107.0493(10)<br>79.0546(5)		7-Hydroxycoumarin
42	5.88*	355.1023	355.1017	-1.71	$C_{16}H_{18}O_9$	MS^2[355]:		Chlorogenic acid

						163.0385(100)<br>193.0457(45)<br>145.0280(11)<br>175.0342(3)<br>95.0331(6)		
42	5.88*	353.0878	353.0877	-0.19	$C_{16}H_{18}O_9$		MS^2[353]:<br>191.0552(100)<br>127.0388(1)<br>173.0447(1)<br>111.0437(1)<br>93.0331(1)	Chlorogenic acid
43	5.99[#]	347.1700	347.1694	-1.68	$C_{16}H_{26}O_8$	MS^2[347]:<br>167.1062(100)<br>137.0957(93)<br>109.1012(50)<br>107.0856(36)<br>121.1011(33)<br>123.0800(13)		Jasminoside B/D/G
44	6.29*	179.0349	179.0340	-5.49	$C_9H_8O_4$		MS^2[179]:<br>135.0439(100)<br>179.0340(9)<br>137.0437(1)<br>87.0074(1)<br>59.0123(1)	Caffeic acid
45	6.51[#]	611.1617	611.1611	-0.93	$C_{27}H_{32}O_{16}$		MS^2[611]:<br>119.0489(100)<br>283.0613(38)<br>295.0608(32)<br>205.0134(51)	Safflomin A

46	6.91#	291.0146	291.0145	-0.48	$C_{13}H_8O_8$		MS^2[291]:<br>175.0390(100)<br>159.0435(11)<br>247.0244(14)<br>203.0341(80)	Brevifolincarboxylic acid
47	7.18#	177.0546	177.0543	-1.70	$C_{10}H_8O_3$	MS^2[177]:<br>149.0594(100)<br>159.0438(5)<br>131.0489(6)<br>105.0700(22)<br>103.0543(16)<br>93.0701(64)<br>79.0546(5)		4-Methylumbelliferone
48	7.19#	551.1963	551.1963	-1.26	$C_{23}H_{34}O_{15}$	MS^2[551]:<br>149.0593(100)<br>209.0801(19)<br>121.0646(33)<br>177.0542(42)		Genipin 1-O-beta-D-Gentiobioside
48	7.19#	549.1824	549.1819	-0.99	$C_{23}H_{34}O_{15}$		MS^2[549]:<br>207.0655(15)<br>123.0438(58)<br>101.0230(100)<br>68.9967(18)<br>147.0439(7)<br>89.0229(7)	Genipin 1-O-beta-D-Gentiobioside
49	7.29#	785.0842	785.0844	0.15	$C_{34}H_{26}O_{22}$		MS^2[785]:<br>300.9987(100)<br>249.0403(42)<br>231.0288(12)<br>275.0197(50)<br>169.0127(22)	Tellimagradin I

50	7.41#	595.1668	595.1670	2.19	$C_{27}H_{32}O_{15}$		MS^2[595]:<br>355.0822(100)<br>256.0244(30)<br>271.0614(11)<br>207.0655(16)<br>313.0715(31)	Isobutrin
51	7.57#	291.0863	291.0858	-1.60	$C_{15}H_{14}O_6$	MS^2[291]:<br>273.0750(68)<br>165.0545(44)<br>147.0440(12)<br>139.0389(80)		Epicatechin
52	7.61#	225.0757	225.0754	-1.24	$C_{11}H_{12}O_5$	MS^2[225]:<br>175.0386(100)<br>207.0648(44)<br>147.0437(66)<br>119.0490(70)<br>91.0545(23)		Sinapic acid
53	7.61*	633.0733	633.0733	-0.06	$C_{27}H_{22}O_{18}$		MS^2[633]:<br>300.9988(100)<br>275.0196(22)<br>169.0132(3)	Corilagin
54	7.81#	167.0704	167.0699	-1.74	$C_9H_{10}O_3$	MS^2[167]:<br>121.0647(100)<br>103.0543(2)<br>93.0701(12)<br>139.1110(10)<br>149.0594(54)		Paeonol
55	7.82#	387.1296	387.1291	-1.22	$C_{17}H_{24}O_{10}$		MS^2[387]:<br>101.0230(100)<br>123.0438(55)<br>68.9967(22)	Geniposide

							207.0654(10)<br>147.0439(9)<br>225.0763(6)	
56	7.83#	225.0768	225.0760	-3.54	$C_{11}H_{14}O_5$		MS^2[225]:<br>101.0230(100)<br>123.0438(28)<br>147.0440(28)	Genipin
57	7.84#	118.0651	118.0651	-0.22	C_8H_7N	MS^2[118]:<br>118.0650(100)<br>95.0493(26)<br>105.0450(7)<br>72.0812(5)		Indole
58	7.88#	209.0808	209.0805	-1.37	$C_{11}H_{12}O_4$	MS^2[209]:<br>121.0646(100)<br>191.0695(15)<br>163.0377(6)<br>119.0484(6)<br>93.0701(15)		Ethyl caffeate
59	7.90#	635.0889	635.0891	0.19	$C_{27}H_{24}O_{18}$		MS^2[635]:<br>169.0132(100)<br>465.0672(2)<br>313.0566(12)<br>125.0231(28)<br>211.0240(17)<br>221.0449(11)	1,2,6-triple-O-galacyl-β -D-glucose
60	7.95#	249.1485	249.1481	-1.49	$C_{15}H_{20}O_3$	MS^2[249]:<br>213.1268(100)<br>231.1371(54)<br>173.0957(35)<br>143.0852(76)		Parthenolide
61	7.95#	153.0546	153.0544	-1.44	$C_8H_8O_3$	MS^2[153]:		4-Hydroxyphenylacetic acid

						109.0440(100)<br>125.0595(61)<br>107.0337(42)<br>91.0051(17)		isomer
62	8.02#	151.0400	151.0398	-7.26	$C_8H_8O_3$		MS^2[151]:<br>133.0153(100)<br>107.0122(27)	4-Hydroxyphenylacetic acid isomer
63	8.20#	249.1485	249.1482	-1.01	$C_{15}H_{20}O_3$	MS^2[249]:<br>143.0852(100)<br>213.1267(72)<br>185.1320(57)<br>157.1008(45)<br>147.1164(47)<br>171.0801(62)		Pterosin A
64	8.28*#	447.0932	447.0930	-0.59	$C_{21}H_{20}O_{11}$		MS^2[449]:<br>327.0507(100)<br>257.0614(43)<br>285.0403(25)<br>151.0021(1)<br>178.9975(1)	Orientin
65	8.29#	565.1551	565.1548	-0.59	$C_{26}H_{28}O_{14}$	MS^2[565]:<br>379.0806(100)<br>397.0904(21)<br>403.0808(8)<br>409.0876(15)		Schaftoside
66	8.34#	169.1223	169.1221	-1.04	$C_{10}H_{16}O_2$	MS^2[169]:<br>85.0651(100)<br>69.0704(48)<br>151.1113(30)<br>109.0649(67)<br>123.1167(84)		Chrysanthemic acid

						128.9505(51)		
67	8.35#	287.0561	287.0559	-0.70	$C_{15}H_{12}O_6$		MS^2[287]: 109.0281(100) 121.0279(8) 135.0076(29) 149.0232(34) 163.0025(27) 225.0550(18) 241.0501(4) 259.0609(5) 269.0456(8)	Dihydrofisetin
68	8.40#	197.0455	197.0446	-4.40	$C_9H_{10}O_5$		MS^2[197]: 197.0446(100) 162.8379(70) 169.0131(79) 140.0101(10) 111.0074(5) 125.0231(46)	Syringic acid
69	8.42#	163.0400	163.0398	-0.55	$C_9H_8O_3$		MS^2[163]: 119.0489(100) 117.2941(1) 162.8379(7)	p-Coumaric acid
70	8.44#	151.0400	151.0398	-7.46	$C_8H_8O_3$		MS^2[151]: 133.0153(100) 107.0125(22)	4-Hydroxyphenylacetic acid isomer
71	8.50#	151.0400	151.0390	-7.06	$C_8H_8O_3$		MS^2[151]: 133.0153(100) 107.0126(5)	4-Hydroxyphenylacetic acid isomer
72	8.52#	183.0651	183.0650	-1.01	$C_9H_{10}O_4$	MS^2[183]: 123.0439(100) 113.9636(52)		4-Hydroxy-3,5-dimethoxybenzaldehyde

						95.0493(95)<br>155.0699(14)<br>140.0464(33)<br>131.0740(30)		
73	8.52[#]	183.0651	183.0650	-1.01	$C_9H_{10}O_4$	MS^2[183]:<br>123.0439(100)<br>113.9636(52)<br>141.9575(7)<br>95.0493(95)		Methyl oxalate
74	8.62*[#]	300.9989	300.9986	-1.30	$C_{14}H_6O_8$		MS^2[300]:<br>300.9986(100)<br>283.9965(3)<br>257.0089(4)<br>229.0135(6)<br>185.0233(3)	Ellagic acid
75	8.65[#]	183.1026	183.1027	0.29	$C_{10}H_{16}O_3$		MS^2[183]:<br>183.1016(100)<br>139.1115(53)<br>157.1222(6)<br>112.9843(3)	Jasminodiol
76	8.69[#]	193.0495	193.0494	-0.44	$C_{10}H_8O_4$	MS^2[193]:<br>193.0492(100)<br>133.0282(32)<br>178.0257(12)<br>165.0543(4)<br>137.0594(5)<br>122.0360(1)<br>107.9598(1)		Scopoletin
77	8.69*[#]	609.1461	609.1461	0.02	$C_{27}H_{30}O_{16}$		MS^2[609]:<br>300.0271(100)<br>301.0348(27)	Rutin

							271.0243(16)<br>151.0027(9)	
78	8.70#	521.1664	521.1662	-0.48	$C_{25}H_{30}O_{12}$		MS^2[521]:<br>145.0282(100)<br>163.0388(56)<br>117.0330(5)<br>71.0123(7)<br>107.0488(26)<br>119.0489(40)<br>125.0594(27)<br>151.0752(39)	2 '(4'-hydroxycinnamoyl) -Polygonatum japonicum glycoside
79	8.75*#	463.0881	463.0880	-0.34	$C_{21}H_{20}O_{12}$		MS^2[463]:<br>300.0271(100)<br>301.0350(18)<br>151.0025(9)	Isoquercitrin
80	8.77*#	431.0983	431.0979	-1.02	$C_{21}H_{20}O_{10}$		MS^2[431]:<br>311.0558(100)<br>283.0609(77)<br>269.0451(8)<br>151.0024(2)	Vitexin
81	8.80#	479.0820	479.0813	-1.43	$C_{21}H_{18}O_{13}$	MS^2[479]:<br>303.0491(100)<br>272.0727(12)<br>113.0233(8)<br>153.0336(4)<br>85.0287(15)		Quercetin3-O- β -D-Glucuronide
82	8.82#	319.1176	319.1169	-2.21	$C_{17}H_{18}O_6$	MS^2[319]:<br>255.1009(100)<br>283.0958(24)<br>301.1063(37)<br>227.1061(21)		Agarotetrol

						164.0464(27)<br>136.0517(1)<br>129.0696(3)<br>105.0700(17)		
83	8.82$^{\#}$	317.1030	317.1026	-1.27	$C_{17}H_{18}O_6$		MS^2[317]:<br>124.0153(28)<br>151.0310(29)<br>269.0469(33)<br>287.0569(28)<br>302.0784(17)	3’-hydroxy-8-methoxyvestitol
84	8.84$^{\#}$	549.1613	549.1610	-0.54	$C_{26}H_{30}O_{13}$	MS^2[573]:<br>419.1385(45)<br>257.0807(22)		Isoliquiritin
85	8.85$^{\#}$	257.0808	257.0802	-2.12	$C_{15}H_{12}O_4$	MS^2[257]:<br>137.0230(100)<br>147.0437(45)<br>239.0697(6)<br>211.0748(7)<br>119.0490(6)		Isoliquiritigenin isomers
86	8.85*$^{\#}$	463.0881	463.0877	-1.06	$C_{21}H_{20}O_{12}$		MS^2[463]:<br>300.0271(100)<br>301.0348(25)<br>271.0245(25)<br>273.0394(5)<br>178.9976(7)<br>151.0025(11)<br>255.0295(12)	Hyperoside
87	8.86*$^{\#}$	417.1191	417.1187	-0.95	$C_{21}H_{22}O_9$		MS^2[417]:<br>255.0659(74)<br>135.0075(100)<br>119.0489(53)	Liquiritin

							153.0181(19)	
88	9.08	595.1657	595.1652	-0.89	$C_{27}H_{30}O_{15}$	MS^2[595]: 287.0543(100) 178.0287(22) 71.0496(16)		Kaempferol-3-O-rutinoside
88	9.08	593.1511	593.1509	-0.43	$C_{27}H_{30}O_{15}$		MS^2[593]: 285.0401(100) 284.0322(99) 255.0294(25) 227.0341(8) 151.0028(3)	Kaempferol-3-O-rutinoside
89	9.09[#]	449.1089	449.1086	-0.66	$C_{21}H_{22}O_{11}$		MS^2[449]: 287.0558(100) 286.0480(88) 181.0132(30) 193.0130(10) 178.9976(3)	Carthamidin-5-glucoside
90	9.11[#]	447.0932	447.0931	-0.32	$C_{21}H_{20}O_{11}$		MS^2[447]: 284.0025(100) 255.0292(63) 227.0340(30) 256.0371(12) 285.0404(23)	Orientin isomer
91	9.18[#]	623.1617	623.1615	-0.41	$C_{28}H_{32}O_{16}$		MS^2[623]: 300.0272(100) 285.0401(92) 256.0295(9) 271.0246(36) 272.0325(25)	Narcissoside
92	9.23[#]	462.0792	462.0797	-0.94	$C_{21}H_{17}O_{12}$	MS^2[463]: 285.0543(100)		Luteolin-7-O- beta-D-glucuronide

						255.0797(21)<br>85.0287(13)<br>113.0233(7)		
93	9.24#	515.1194	515.1190	-0.93	$C_{25}H_{24}O_{12}$		MS^2[515]:<br>191.0551(100)<br>179.0340(23)<br>135.0439(9)	Isochlorogenic acid B
94	9.26#	447.0932	447.0930	-0.46	$C_{21}H_{20}O_{11}$		MS^2[447]:<br>284.0323(100)<br>285.0401(29)<br>255.0295(63)<br>227.0344(27)<br>256.0372(10)	Cynaroside
95	9.27#	371.1125	371.1118	-1.83	$C_{20}H_{18}O_7$	MS^2[371]:<br>371.1086(100)<br>165.1264(49)<br>175.0385(22)<br>191.1411(23)<br>91.0575(33)<br>137.0948(18)<br>136.1110(28)		Uralenol
96	9.31#	565.1926	565.1926	-0.06	$C_{27}H_{34}O_{13}$		MS^2[565]:<br>205.0498(100)<br>223.0606(90)<br>265.0715(47)<br>325.0928(8)<br>164.0468(50)<br>221.0812(53)	11-(6-O-trans-sinapoyl-glucopyranosyl) gardendiol
97	9.36#	599.0678	599.0679	0.12	$C_{27}H_{20}O_{16}$		MS^2[599]:<br>300.9987(100)<br>169.0130(16)	4-O-(4’-O-galloyl-rhamnosyl) ellagic acid

							125.0228(6)	
98	9.38#	315.0146	315.0145	-0.54	$C_{15}H_8O_8$		MS^2[315]:<br>299.9908(100)<br>271.0463(27)<br>300.9984(13)<br>227.0486(6)	3-O-methylellagic acid isomer
99	9.38#	269.0455	269.0454	-0.51	$C_{15}H_{10}O_5$		MS^2[269]:<br>133.0282(89)<br>135.0074(21)<br>241.0506(3)<br>269.0452(100)	Genistein isomer
100	9.39#	491.0831	491.0828	-0.46	$C_{22}H_{20}O_{13}$		MS^2[491]:<br>300.0272(100)<br>345.0508(61)<br>271.0247(19)<br>255.0295(12)<br>151.0027(2)	Isorhamnetin 3-glucuronide
100	9.40#	493.0976	493.0971	-1.01	$C_{22}H_{20}O_{13}$	MS^2[493]:<br>347.0648(100)<br>85.0287(13)<br>257.0233(6)<br>273.0467(1)		Isorhamnetin 3-glucuronide
101	9.43#	565.1562	565.1560	-0.37	$C_{26}H_{30}O_{14}$		MS^2[565]:<br>271.0611(100)<br>151.0025(66)<br>227.0706(27)	Cassiaside B
102	9.46#	179.0702	179.0700	-1.23	$C_{10}H_{10}O_3$	MS^2[179]:<br>147.0437(100)<br>119.0491(46)<br>91.0545(16)<br>133.0646(26)		6,7-dihydroxyindan-4-carbaldehyde

						105.0700(21)		
103	9.48#	515.1194	515.1190	-0.93	$C_{25}H_{24}O_{12}$		MS^2[515]:<br>173.0445(100)<br>179.0339(75)<br>191.0552(35)<br>135.0439(28)<br>353.0871(1)	3,5-di-caffeoylquinic acid
104	9.57#	147.0440	147.0437	-1.81	$C_9H_6O_2$	MS^2[147]:<br>119.0490(100)<br>91.0545(33)<br>65.0391(1)		Coumarin
105	9.59#	137.0244	137.0231	-9.03	$C_7H_6O_3$		MS^2[137]:<br>93.0331(100)<br>137.0231(38)<br>136.8618(7)<br>94.0365(1)	3-Hydroxybenzoic acid
106	9.61#	315.0146	315.0145	-0.16	$C_{15}H_8O_8$		MS^2[315]:<br>300.9989(100)<br>271.0463(27)<br>227.0489(6)<br>300.9942(3)	3-O-methylellagic acid
107	9.62#	269.0455	269.0454	-0.40	$C_{15}H_{10}O_5$		MS^2[269]:<br>269.0454(100)<br>225.0552(82)<br>241.0499(26)<br>197.0603(14)	Emodin
108	9.76#	167.0349	167.0339	-6.05	$C_8H_8O_4$		MS^2[167]:<br>167.0338(100)<br>152.0103(36)<br>111.0073(12)	Vanillic acid isomer
109	9.77#	419.1336	419.1331	-1.29	$C_{21}H_{22}O_9$	MS^2[419]:		Liquiritin isomer

						257.0802(100)<br>137.0230(68)<br>147.0437(39)<br>119.0492(2)<br>239.0697(9)		
110	9.81#	419.0983	419.0983	2.59	$C_{20}H_{20}O_{10}$		MS^2[419]:<br>257.0451(100)<br>213.0549(3)	Cassiaside
111	9.82#	301.0706	301.0703	-1.18	$C_{16}H_{12}O_6$	MS^2[301]:<br>301.0700(100)<br>286.0465(21)<br>257.0436(2)<br>213.0537(11)		Tectorigenin
112	9.86*#	193.0506	193.0497	-4.47	$C_{10}H_{10}O_4$		MS^2[193]:<br>193.0497(100)<br>149.0596(37)<br>178.0259(23)<br>134.0361(20)	Ferulic acid
113	9.88#	549.1613	549.1611	-0.43	$C_{26}H_{30}O_{13}$	MS^2[551]:<br>257.0801(100)<br>137.0230(68)<br>147.0437(41)<br>239.0697(10)<br>213.0748(7)<br>242.0566(3)<br>163.0385(3)		Isoliquiritin apioside isomer
114	9.93#	491.1194	491.1192	1.69	$C_{23}H_{24}O_{12}$		MS^2[491]:<br>313.0353(100)<br>298.0115(42)<br>329.0682(4)<br>270.0170(30)	Aurantio-obtusin-6-O- β -D-glucoside

115	9.96#	559.1457	559.1452	-0.81	$C_{27}H_{28}O_{13}$		242.0204(7)<br>285.0402(52)<br>MS2[559]:<br>173.0444(100)<br>223.0605(16)<br>164.0467(9)<br>137.0231(5)	4-O-sinapoyl-5-O-caffeoylquninic acid
84	10.07*#	419.1336	419.1329	-1.64	$C_{21}H_{22}O_9$	MS2[419]:<br>257.0801(100)<br>137.0438(63)<br>147.0436(38)<br>239.0697(10)<br>211.0747(8)<br>119.0492(2)		Isoliquiritin
116	10.09#	301.0717	301.0715	-0.57	$C_{16}H_{14}O_6$		MS2[301]:<br>121.0283(3)<br>135.0076(4)<br>153.0179(2)<br>177.0183(70)<br>268.0374(100)	Homoeriodictyol
117	10.16#	269.0444	269.0438	-2.12	$C_{15}H_8O_5$	MS2[269]:<br>269.0802(100)<br>213.0902(9)<br>254.0567(11)<br>237.0539(5)<br>118.0411(2)<br>253.0492(3)		Coumestrol
118	10.18#	431.1336	431.1330	-1.32	$C_{22}H_{22}O_9$	MS2[431]:<br>269.0800(100)<br>254.0565(2)		Ononin
119	10.18#	285.0404	285.0402	-0.85	$C_{15}H_{10}O_6$		MS2[285]:	Luteolin

							150.0310(100)<br>285.0757(2)<br>270.0530(18)<br>257.0084(8)	
120	10.20[#]	253.0506	253.0502	-1.63	$C_{15}H_{10}O_4$		MS^2[253]:<br>117.0332(31)<br>135.0074(16)<br>225.0550(1)<br>253.0529(100)	Chrysophanic acid
121	10.30*[#]	285.0404	285.0403	-0.43	$C_{15}H_{10}O_6$		MS^2[285]:<br>285.0403(100)<br>257.0448(10)<br>229.0497(3)<br>135.0440(4)	Kaempferol
122	10.32*[#]	255.0662	255.0658	-1.54	$C_{15}H_{12}O_4$		MS^2[255]:<br>119.0489(100)<br>135.0075(40)<br>153.0181(17)<br>255.0659(3)<br>91.0175(14)	Liquiritigenin
122	10.33*[#]	257.0808	257.0804	-1.42	$C_{15}H_{12}O_4$	MS^2[257]:<br>137.0230(100)<br>147.0437(46)<br>117.0334(3)<br>119.0491(7)<br>257.0802(20)<br>211.0748(7)<br>239.0697(5)		Liquiritigenin
123	10.39[#]	287.0561	287.0559	-0.70	$C_{15}H_{12}O_6$		MS^2[287]:<br>135.0438(100)<br>151.0025(68)	Eriodictyol

							107.0124(19)<br>65.0018(6)<br>287.0562(1)<br>257.0084(6)	
124	10.43*#	303.0499	303.0495	-1.42	$C_{15}H_{10}O_7$	MS^2[303]:<br>303.0492(100)<br>153.0180(5)<br>165.0180(4)<br>229.0489(6)<br>257.0438(5)<br>201.0549(2)		Quercetin
125	10.46#	477.1038	477.1034	-0.75	$C_{22}H_{22}O_{12}$		MS^2[477]:<br>314.0429(100)<br>299.0193(91)<br>271.0246(14)<br>285.0404(3)<br>165.0182(18)	Isorhamnetin-3-O-glucoside
126	10.47#	303.0499	303.0494	-1.71	$C_{15}H_{10}O_7$	MS^2[303]:<br>285.1113(100)<br>194.0571(25)<br>187.0283(2)		Quercetin isomer
124	10.47*#	301.0353	301.0351	-0.82	$C_{15}H_{10}O_7$		MS^2[301]:<br>151.0025(100)<br>135.0439(18)<br>121.0281(25)<br>119.0282(2)<br>178.9976(31)	Quercetin
127	10.54#	263.1288	263.1287	-0.62	$C_{15}H_{20}O_4$		MS^2[263]:<br>204.1149(100)<br>201.1278(45)<br>163.0755(30)	Abscisic acid

							219.1381(78)<br>151.0735(56)	
128	10.59#	285.0757	285.0751	-2.07	$C_{16}H_{12}O_5$	MS^2[285]:<br>285.0749(100)<br>270.0514(56)<br>253.0488(18)<br>225.0540(16)<br>242.0569(1)		Genkwanin
129	10.68#	285.0768	285.0766	-0.66	$C_{16}H_{14}O_5$		MS^2[285]:<br>135.0075(100)<br>134.0363(3)<br>148.0154(4)<br>270.0531(32)	Sakuranetin
130	10.79#	313.0717	313.0715	-0.74	$C_{17}H_{14}O_6$		MS^2[313]:<br>283.0244(100)<br>298.0477(24)<br>313.0718(5)<br>255.0293(12)	Pectolinarigenin
131	10.81*#	315.0510	315.0509	-0.40	$C_{16}H_{12}O_7$		MS^2[315]:<br>300.0272(100)<br>271.0247(46)<br>255.0296(23)<br>243.0290(5)<br>107.0211(2)	Isorhamnetin
132	10.85#	249.1485	249.1481	-1.49	$C_{15}H_{20}O_3$	MS^2[249]:<br>137.0549(100)<br>143.0852(34)<br>231.1375(15)<br>213.1269(33)<br>203.1425(11)<br>157.1007(37)		Santamarine

133	10.90#	337.0837	337.0831	-1.77	$C_{17}H_{17}O_5Cl$	MS^2[337]:<br>91.0544(100)<br>319.0723(81)<br>301.0618(66)<br>283.0957(55)<br>265.0852(93)<br>255.1008(20)		8-Chloro-2-(2-phenylethyl)-5,6,7-trihydroxy-5,6,7,8-tetrahydrochromone
134	10.90#	537.2330	537.2324	-1.04	$C_{27}H_{36}O_{11}$	MS^2[537]:<br>175.0386(100)<br>207.0648(69)<br>119.0491(21)<br>123.1168(29)		6’-O-trans-sinapoyl jasminoside A
135	10.93#	225.1132	225.1126	-2.76	$C_{12}H_{18}O_4$		MS^2[225]:<br>207.1018(100)<br>181.1223(31)<br>178.8329(18)<br>163.1117(18)	Senkyunolide
136	10.96#	507.1133	507.1128	-0.92	$C_{23}H_{22}O_{13}$	MS^2[507]:<br>331.0804(100)<br>316.0569(16)<br>85.0287(14)		3,4,3′-trimethylated ellagic acid-4’-O-beta-D-glucoside
137	10.98#	855.4008	855.4001	-0.87	$C_{42}H_{62}O_{18}$	MS^2[855]:<br>141.0178(100)<br>233.1532(54)<br>187.1478(93)<br>215.1426(41)<br>237.1466(14)<br>145.1003(18)<br>205.1581(21)		Glycyrrhizin G2
138	11.08#	219.1743	219.1740	-1.24	$C_{15}H_{22}O$	MS^2[219]:<br>201.1633(100)		Germacrone

						219.1737(48)<br>159.1165(60)<br>145.1008(92)<br>161.1321(26)		
139	11.13*#	271.0600	271.0596	-1.51	$C_{15}H_{10}O_5$	MS^2[269]:<br>271.0594(95)<br>215.0700(2)<br>153.0179(2)<br>243.0644(2)<br>229.0854(14)<br>253.0856(4)		Genistein
140	11.14*#	271.0611	271.0609	-0.95	$C_{15}H_{12}O_5$		MS^2[271]:<br>151.0025(100)<br>178.0183(11)<br>119.0489(80)<br>107.0124(32)	Naringenin
141	11.18#	179.0338	179.0335	-1.76	$C_9H_6O_4$	MS^2[179]:<br>95.0493(100)<br>123.0439(86)<br>151.0386(63)<br>133.0294(2)<br>105.0336(14)		Esculetin
119	11.24#	287.0550	287.0908	-1.92	$C_{15}H_{10}O_6$	MS^2[287]:<br>153.0542(100)<br>269.0799(11)<br>258.0582(6)<br>213.0893(1)<br>165.0537(1)<br>147.0437(4)		Luteolin
142	11.33#	345.0979	345.0977	-0.74	$C_{18}H_{18}O_7$		MS^2[345]:<br>151.0389(100)	5,7-dihydroxy-2’,3’,4’-trimethoxy isoflavanone

							135.0074(95)<br>177.0183(69)<br>295.0615(21)<br>315.0506(20)	
143	11.35#	315.0510	315.0509	-0.30	$C_{16}H_{12}O_7$		MS^2[315]:<br>271.0246(100)<br>300.0271(51)<br>243.0296(46)<br>165.9898(29)<br>219.8455(10)	Isorhamnetin isomer
144	11.46#	283.0611	283.0609	-1.01	$C_{16}H_{12}O_5$		MS^2[283]:<br>283.0609(33)<br>268.0374(100)<br>240.0424(3)<br>211.0394(1)<br>135.0075(5)	Wogonin
145	11.52#	514.2843	514.2835	-1.72	$C_{26}H_{45}NO_7S$		MS^2[514]:<br>515.2839(100)<br>124.0062(2)	Cholaic acid
146	11.59#	301.0706	301.0701	-1.78	$C_{16}H_{12}O_6$	MS^2[301]:<br>286.0465(100)<br>301.0699(35)<br>257.0436(6)<br>213.0542(1)		Tectorigenin isomer
147	11.61#	269.0819	269.0816	-0.97	$C_{16}H_{14}O_4$		MS^2[269]:<br>135.0438(13)<br>226.0629(13)<br>254.0580(48)	2'-methoxyisoliquiritigenin
148	11.77#	329.0666	329.0666	-0.08	$C_{20}H_{22}O_4$		MS^2[329]:<br>314.0426(10)<br>299.0198(87)	Aurantio-obtusin isomer

							285.0405(5)<br>271.0247(8)<br>243.0295(7)<br>153.0182(43)	
149	11.78*	257.0808	257.0803	-1.89	$C_{15}H_{12}O_4$	MS^2[257]:<br>137.0229(100)<br>147.0436(46)<br>119.0490(6)<br>257.0801(19)<br>81.0338(1)		Isoliquiritigenin
150	11.78*	255.0662	255.0658	-1.62	$C_{15}H_{12}O_4$		MS^2[255]:<br>119.0498(100)<br>135.0074(42)<br>153.0181(17)<br>255.0658(5)<br>91.0174(14)	Isoliquiritigenin
150	11.84#	573.2341	573.2336	-0.86	$C_{30}H_{38}O_{11}$		MS^2[573]:<br>59.0124(100)<br>111.0438(5)<br>489.2086(26)<br>425.1946(8)<br>531.2230(4)	Isotoosendanin
151	11.95#	329.2333	329.2331	-0.69	$C_{18}H_{34}O_5$		MS^2[329]:<br>171.1016(100)<br>201.1126(2)<br>293.2103(4)<br>311.2222(3)	9(S),10(S),11(R)-trihydroxy-12(Z)-octadecenoic acid
152	11.98#	258.1488	258.1484	-1.49	$C_{16}H_{19}NO_2$	MS^2[258]:<br>173.0593(100)<br>107.0492(12)<br>145.0644(10)		Medifoxamine

						152.1066(6)	
153	12.05#	299.0561	299.0558	-0.77	$C_{16}H_{12}O_6$	MS²[299]: 284.0323(100) 299.0558(50) 257.0657(62) 216.0420(22) 135.0046(16)	Hydroxygenkwanin
154	12.10#	267.0662	267.0659	-1.13	$C_{16}H_{12}O_4$	MS²[267]: 252.0423(100) 267.0659(23) 208.0523(1) 195.0442(1) 132.0204(2) 91.0184(1)	Formononetin
155	12.11#	271.0975	271.0971	-1.63	$C_{16}H_{16}O_4$	MS²[271]: 135.0439(100) 109.0281(63) 123.0438(16) 149.0596(28) 197.0597(5) 241.0499(8) 256.0752(10)	Vestitol
156	12.11#	573.2341	573.2334	-1.18	$C_{30}H_{38}O_{11}$	MS²[573]: 531.2230(62) 489.2099(11) 453.1921(8) 425.1990(3) 111.0437(3)	Toosendanin
157	12.18#	329.0666	329.0663	-1.11	$C_{17}H_{14}O_7$	MS²[329]: 299.0193(100) 314.0429(41)	Aurantio-obtusin

							285.0401(14)<br>271.0245(19)	
158	12.18#	329.0666	329.0663	-1.11	$C_{17}H_{14}O_7$		MS2[329]:<br>299.0193(100)<br>314.0429(41)<br>243.0293(3)	Cirsiliol
159	12.26#	248.1281	248.1276	-1.85	$C_{14}H_{17}NO_3$	MS2[248]:<br>175.0385(100)<br>192.0649(2)<br>145.0280(20)		Fagaramide
160	12.27#	503.3378	503.3374	-0.70	$C_{30}H_{48}O_6$		MS2[503]:<br>409.3111(100)<br>421.3103(35)<br>453.3009(36)<br>457.3327(19)<br>473.3250(9)<br>485.3261(6)	Arjugenin
161	12.30#	823.4110	823.4096	-1.76	$C_{42}H_{62}O_{16}$	MS2[823]:<br>453.3348(91)<br>471.3443(10)<br>217.1580(66)		Glycyrrhizic acid
162	12.30#	821.3965	821.3960	-0.55	$C_{42}H_{62}O_{16}$		MS2[821]:<br>113.0230(100)<br>193.0344(32)<br>351.0567(12)<br>75.0073(35)<br>85.0280(50)	Glycyrrhizic acid
162	12.33#	239.0713	239.0708	-2.29	$C_{15}H_{12}O_3$		MS2[239]:<br>197.0598(82)<br>135.0075(15)<br>153.0182(15)	2’,4’-dihydroxychalcone

163	12.35#	329.1030	329.1028	-0.76	$C_{18}H_{18}O_6$		MS^2[329]: 135.0075(100) 148.0154(3) 161.0233(23) 284.0325(14) 299.0557(83) 314.0795(13)	3'-O-methylviolanone
164	12.52#	135.0804	135.0801	-2.16	$C_9H_{10}O$	MS^2[135]: 107.0492(100) 89.0794(17)		Cinnamic alcohol
165	12.52#	299.0924	299.0921	-1.13	$C_{17}H_{16}O_5$		MS^2[299]: 135.0074(100) 161.0232(8) 241.0508(1) 269.0449(18) 283.0608(6)	Methylnissolin
166	12.62#	369.1332	369.1325	-1.96	$C_{21}H_{20}O_6$	MS^2[369]: 301.0698(100) 213.0697(66) 257.1322(10) 271.0879(13) 285.0749(21)		Glycycoumarin
167	12.65#	272.1281	272.1275	-2.06	$C_{16}H_{17}NO_3$	MS^2[272]: 201.0541(100) 135.0437(39) 98.0602(10) 171.0435(10)		Piperyline
168	12.77	651.2658	651.2658	-0.03	$C_{32}H_{44}O_{14}$		MS^2[651]: 239.1798(100) 283.1703(32)	Crocin III
169	12.83#	318.1335	318.1330	-1.88	$C_{17}H_{19}NO_5$	MS^2[318]:		Piperodione

						205.0490(100)<br>233.0439(1)<br>168.1015(9)<br>149.0229(19)<br>196.0964(1)		
170	12.91#	283.0611	283.0610	-0.45	$C_{16}H_{12}O_5$		MS^2[283]:<br>240.0423(100)<br>212.9935(2)<br>283.0609(61)	Physcione
171	12.97#	267.1015	267.1010	-1.99	$C_{17}H_{14}O_3$	MS^2[267]:<br>267.1008(100)<br>176.0464(62)<br>91.0545(89)<br>137.0229(2)<br>110.0362(1)		Benzarone
172	13.09#	355.1176	355.1170	-1.73	$C_{20}H_{18}O_6$	MS^2[355]:<br>151.0386(100)<br>179.0334(84)<br>189.0905(29)<br>213.0906(37)<br>257.1067(9)		Glycyrrhiza isoflavanone
173	13.10#	255.0662	255.0659	-1.38	$C_{15}H_{12}O_4$		MS^2[255]:<br>255.0658(100)<br>213.0549(21)<br>151.0025(20)<br>211.0756(7)<br>107.0124(9)	Isoliquiritigenin isomers
174	13.17#	274.1437	274.1430	-2.66	$C_{16}H_{19}NO_3$	MS^2[274]:<br>201.0541(100)<br>135.0437(95)<br>171.0436(12)		Piperlonguminine

						143.0487(14)		
175	13.18#	391.2842	391.2830	-3.08	$C_{24}H_{38}O_4$	MS^2[391]:<br>213.1632(100)<br>355.2622(86)<br>145.1007(56)		12-ketolcholic acid
176	13.24#	283.0611	283.0608	-1.12	$C_{16}H_{12}O_5$		MS^2[283]:<br>268.0373(100)<br>240.0424(1)<br>224.0471(1)<br>211.0390(1)<br>135.0075(2)	Pea chalcone B
177	13.24#	283.0611	283.0608	-1.12	$C_{16}H_{12}O_5$		MS^2[283]:<br>268.0373(100)<br>240.0424(1)<br>224.0471(1)<br>211.0390(1)<br>195.0440(2)	Melanettin
178	13.28#	203.1794	203.1792	-1.12	$C_{15}H_{22}$	MS^2[203]:<br>81.0702(47)<br>95.0857(79)<br>109.1012(63)<br>147.1164(67)<br>203.1790(100)		α-curcumene
179	13.28#	353.1383	353.1377	-1.59	$C_{21}H_{20}O_5$	MS^2[353]:<br>193.0492(100)<br>173.0958(25)<br>297.0742(20)<br>353.1373(18)		Gancaonin M
180	13.29#	327.1590	327.1585	-1.76	$C_{20}H_{22}O_4$	MS^2[327]:<br>137.0594(68)<br>151.0750(100)		Machilin A

						171.0800(8)<br>203.1062(29)	
181	13.29#	345.1696	345.1690	-1.68	$C_{20}H_{24}O_5$	MS^2[345]:<br>137.0594(100)<br>153.0543(20)<br>188.0826(4)<br>165.0907(27)<br>133.0645(20)<br>125.0595(15)<br>105.0700(10)<br>221.1167(13)<br>203.106(11)	Fragransin A2
182	13.37#	369.1332	369.1324	-2.13	$C_{21}H_{20}O_6$	MS^2[369]:<br>285.0748(100)<br>213.0697(68)<br>313.1424(7)<br>271.0593(14)<br>257.0514(25)<br>243.0644(23)	Glycycoumarin isomer
183	13.45#	355.1540	355.1534	-1.66	$C_{21}H_{22}O_5$	MS^2[355]:<br>299.0907(100)<br>153.0543(37)<br>257.0437(27)<br>191.1062(19)<br>178.0620(19)<br>271.0960(12)	Gancaonin I
184	13.52#	311.1277	311.1269	-2.69	$C_{19}H_{18}O_4$	MS^2[311]:<br>220.0724(100)<br>205.0490(8)<br>181.0486(1)<br>311.1268(76)	Tanshinaldehyde

185	13.54#	288.1594	288.1586	-2.64	$C_{17}H_{21}NO_3$	MS^2[288]:<br>135.0437(100)<br>161.0593(5)<br>203.0698(2)<br>112.0757(7)		Piperanine
186	13.55#	343.0823	343.0819	-1.04	$C_{18}H_{16}O_7$		MS^2[343]:<br>313.0351(100)<br>285.0401(22)<br>270.0168(6)<br>242.0217(2)<br>298.0115(14)	Obtusin
187	13.60#	355.1176	355.1167	-2.58	$C_{20}H_{18}O_6$	MS^2[355]:<br>69.0704(100)<br>287.0542(83)<br>257.0320(37)<br>271.0593(16)<br>213.0644(13)		Glycyrrhiza isoflavanone isomer
188	13.87	193.0859	193.0855	-1.71	$C_{11}H_{12}O_3$	MS^2[193]:<br>105.0699(100)<br>133.0644(79)<br>118.0412(16)<br>115.0541(11)<br>79.0546(18)<br>165.0905(51)<br>150.0671(32)<br>137.0593(26)		Myristicin
189	13.94#	455.3519	455.3512	-1.63	$C_{30}H_{46}O_3$	MS^2[455]:<br>189.1633(100)<br>201.1632(40)<br>95.0857(74)<br>409.2362(7)		Wilforlide A

						149.1322(67)<br>177.1633(40)		
190	13.94#	337.1445	337.1444	-0.19	$C_{21}H_{22}O_4$		MS^2[337]:<br>337.1445(100)<br>305.1180(40)<br>187.0755(28)<br>108.0203(44)<br>93.0332(59)	Licochalcone A
191	14.06#	163.0753	163.0750	-1.82	$C_{10}H_{10}O_2$	MS^2[163]:<br>103.0543(100)<br>107.0492(63)<br>133.0489(45)<br>135.0802(69)		Safrol
192	14.06#	397.1586	397.1614	2.65	$C_{30}H_{20}O$	MS^2[397]:<br>216.0753(100)<br>217.0814(1)<br>201.0519(16)		Tetrahydropyranthron
193	14.08#	367.1176	367.1170	-1.59	$C_{21}H_{18}O_6$	MS^2[367]:<br>311.0540(100)<br>281.0436(48)<br>296.0307(47)<br>337.0689(3)<br>283.0584(28)<br>309.0701(11)		Glycyrol
194	14.17#	251.1066	251.1060	-2.37	$C_{17}H_{14}O_2$	MS^2[251]:<br>91.0544(100)<br>160.0515(28)<br>251.1059(84)<br>173.0593(2)		2- (2-phenylethyl) chromone
195	14.20#	372.1805	372.1798	-1.83	$C_{21}H_{22}O_5$	MS^2[372]:<br>86.0968(100)		Gancaonin I isomer

						257.0436(31)<br>149.0230(86)<br>241.0854(25)		
196	14.43#	265.1223	265.1218	-1.57	$C_{18}H_{16}O_2$	MS^2[265]:<br>219.1134(100)<br>128.0618(22)<br>159.0437(44)<br>103.0700(20)<br>143.0489(42)		Cinnamyl cinnamate
197	14.46#	335.0924	335.0924	-0.08	$C_{20}H_{16}O_5$		MS^2[335]:<br>335.0923(100)<br>291.1023(32)<br>135.0079(7)<br>91.0329(4)	Glabrone
198	14.47#	281.1172	281.1165	-2.39	$C_{18}H_{16}O_3$	MS^2[281]:<br>190.0620(93)<br>281.1166(100)<br>91.0545(58)<br>175.0385(1)<br>151.0384(2)<br>147.0438(1)<br>124.0515(1)		Ipriflavone
199	14.54#	312.1594	312.1587	-2.05	$C_{19}H_{21}NO_3$	MS^2[312]:<br>169.0644(100)<br>141.0695(44)<br>131.0489(18)<br>135.0911(3)		Piperine
200	14.62*#	325.1434	325.1428	-1.89	$C_{20}H_{20}O_4$	MS^2[325]:<br>123.0439(100)<br>149.0594(45)<br>189.0905(59)		Glabridin

						137.0800(15)<br>203.1062(4)<br>121.0647(5)		
200	14.62*#	323.1288	323.1286	-0.78	$C_{20}H_{20}O_4$		MS^2[323]:<br>135.0439(100)<br>201.0911(94)<br>323.1286(27)<br>175.0752(24)<br>187.1115(7)<br>91.0538(5)	Glabridin
201	14.69#	314.1750	314.1744	-2.01	$C_{19}H_{23}NO_3$	MS^2[314]:<br>135.0437(100)<br>229.0846(4)<br>179.1300(24)<br>112.0757(72)		Piperdardine
202	14.88	233.1536	233.1531	-1.87	$C_{15}H_{20}O_2$	MS^2[233]:<br>187.1476(100)<br>215.1425(17)<br>145.1008(40)<br>131.0852(34)<br>105.0699(26)<br>81.0702(26)		Isoalantolactone
203	14.88	233.1536	233.1531	-1.87	$C_{15}H_{20}O_2$	MS^2[233]:<br>187.1476(100)<br>215.1425(17)<br>159.1163(20)<br>145.1008(40)<br>131.0852(34)<br>119.0854(14)<br>105.0699(26)<br>91.0545(9)		Costunolide

						81.0702(26)		
204	14.92#	357.1707	357.1705	-0.69	$C_{21}H_{26}O_5$		MS^2[357]:<br>109.0282(100)<br>135.0431(7)<br>245.1532(1)<br>247.1335(73)<br>313.1815(14)	Malabaricone C
205	14.97#	165.0910	165.0907	-1.86	$C_{10}H_{12}O_2$	MS^2[165]:<br>165.0906(100)<br>150.0672(39)<br>122.0728(4)<br>133.0644(57)<br>105.0699(41)<br>118.0413(15)		P-Hydroxyphenyl butanone
206	14.97#	195.1015	195.1012	-1.75	$C_{11}H_{14}O_3$	MS^2[195]:<br>195.1018(100)<br>180.0929(73)<br>167.0699(26)<br>154.0620(12)<br>135.0801(16)<br>113.9783(6)		Methoxyeugenol
207	15.14#	231.1379	231.1376	-1.41	$C_{15}H_{18}O_2$	MS^2[231]:<br>185.1319(100)<br>143.0851(43)<br>213.1268(10)<br>171.0800(7)<br>105.0699(19)<br>91.0544(7)		Lindenenol
208	15.14#	231.1379	231.1376	-1.41	$C_{15}H_{18}O_2$	MS^2[231]:<br>185.1319(100)<br>195.1163(16)		Dehydro-α-curcumene

						213.1268(10)<br>175.0749(10)<br>157.1007(38)<br>143.0851(43)<br>128.0696(15)	
209	15.14#	328.1907	328.1900	-2.01	$C_{20}H_{25}NO_3$	MS^2[328]:<br>131.0489(100)<br>135.0436(62)<br>161.0593(28)<br>229.1210(10)<br>187.0753(16)	Piperlongumine A
210	15.26#	165.0910	165.0908	-1.19	$C_{10}H_{12}O_2$	MS^2[328]:<br>137.0593(100)<br>119.0356(11)	Eugenol isomer
211	15.30#	293.1747	293.1742	-1.76	$C_{17}H_{24}O_4$	MS^2[293]:<br>293.2102(7)<br>95.0493(100)<br>81.0702(13)<br>93.0701(11)<br>79.0546(9)<br>67.0547(9)	9-Acetoxyfukinanolide
212	15.35	224.2008	224.2004	-2.10	$C_{14}H_{25}NO$	MS^2[224]:<br>224.2002(100)<br>168.1378(27)<br>151.1113(17)<br>69.0704(44)<br>81.0338(25)	Pellitorine
213	15.36#	165.0910	165.0908	-1.19	$C_{10}H_{12}O_2$	MS^2[165]:<br>137.0593(100)<br>119.0356(1)	Eugenol isomer
214	15.47#	330.2063	330.2055	-2.51	$C_{20}H_{27}NO_3$	MS^2[330]:	Pipercallosine

215	15.55#	233.1536	233.1531	-1.83	$C_{15}H_{20}O_2$	135.0437(100)<br>229.1217(28)<br>330.3356(47)<br>161.0592(16)<br>MS^2[233]:<br>105.0699(100)<br>215.1424(23)<br>177.0904(17)<br>145.1007(32)<br>151.0750(49)		Atractylenolide
216	15.58	325.1445	325.1443	-0.56	$C_{20}H_{22}O_4$		MS^2[325]:<br>310.1209(100)<br>293.1182(25)<br>254.0580(43)	Dehydrodiisoeugenol
217	15.71	327.1590	327.1584	-1.94	$C_{20}H_{22}O_4$	MS^2[327]:<br>137.0593(100)<br>188.0827(91)<br>171.0799(54)<br>203.1061(32)<br>177.0905(16)		Dehydrodiisoeugenol
217	15.71#	340.1907	340.1899	-2.21	$C_{21}H_{25}NO_3$	MS^2[340]:<br>112.0757(100)<br>131.0489(50)<br>135.0437(22)<br>255.1015(5)<br>227.1068(11)<br>213.0905(7)		Piptigrine
218	15.97#	221.1899	221.1895	-1.82	$C_{15}H_{24}O$	MS^2[221]:<br>81.0702(100)<br>222.0196(73)<br>95.0857(24)		Spathulenol

						111.0137(79)		
219	15.98#	236.2008	236.2004	-1.99	$C_{15}H_{25}NO$	MS^2[236]:<br>112.0757(100)<br>86.0967(59)<br>109.1009(8)<br>151.1112(12)<br>135.1014(4)<br>69.0704(40)		Neopellitorine B
220	16.08#	342.2063	342.2056	-2.25	$C_{21}H_{27}NO_3$	MS^2[342]:<br>135.0437(100)<br>229.1218(14)<br>199.1113(7)<br>86.0967(52)<br>112.0757(27)		Pipernonaline
221	16.13	277.2173	277.2170	-0.84	$C_{18}H_{30}O_2$		MS^2[277]:<br>241.7925(23)<br>134.8934(57)<br>233.1906(11)<br>192.9884(11)<br>59.0122(14)	α-linolenic acid
222	16.18#	209.1172	209.1168	-1.92	$C_{12}H_{16}O_3$	MS^2[209]:<br>194.0933(100)<br>178.0984(90)<br>168.0777(44)<br>153.0542(11)<br>151.0750(22)<br>121.0647(15)<br>163.0749(21)		Isoelemicine
223	16.18#	209.1172	209.1168	-1.92	$C_{12}H_{16}O_3$	MS^2[209]:<br>194.0933(100)<br>178.0984(90)		α-Asarone

						181.0854(95)<br>209.1167(44)	
224	16.27[#]	179.1066	179.1063	-1.54	$C_{11}H_{14}O_2$	MS^2[179]:<br>151.0750(100)<br>138.0671(16)<br>123.0517(7)<br>164.0828(21)<br>179.1060(29)<br>136.0516(16)	Methyleugenol
225	16.32[#]	300.2897	300.2891	-0.54	$C_{18}H_{34}O_2$	MS^2[300]<br>83.0858(100)<br>109.1007(29)<br>97.1013(90)	Oleic acid
226	16.55[#]	344.2220	344.2214	-1.69	$C_{21}H_{29}NO_3$	MS^2[344]:<br>135.0437(100)<br>112.0756(25)<br>161.0590(2)<br>222.1848(4)<br>86.0967(49)	Piperolein B
227	16.88[#]	279.1590	279.1586	-1.74	$C_{16}H_{22}O_4$	MS^2[279]:<br>149.0229(100)<br>156.0533(2)<br>90.0545(2)<br>226.0546(53)	Mansonone N
228	16.98[#]	279.1590	279.1585	-1.85	$C_{16}H_{22}O_4$	MS^2[279]:<br>149.0229(100)<br>279.0925(50)<br>219.0563(33)	Dibutyl phthalate
229	17.14[#]	252.2321	252.2317	-1.63	$C_{16}H_{29}NO$	MS^2[252]:<br>57.0705(100)<br>196.1691(12)	N-isobutyl1-2,4-dodecadienamide

						252.2317(53)<br>95.0857(33)		
230	17.25#	325.1420	325.1429	-1.62	$C_{18}H_{22}O_4$	MS^2[325]:<br>135.0437(100)<br>188.0825(87)<br>171.0800(65)<br>143.0853(57)<br>149.0593(47)		Nordihydroguaiaretic acid
231	17.36#	469.3323	469.3319	-0.77	$C_{30}H_{46}O_4$		MS^2[469]:<br>469.3316(100)<br>425.3410(12)<br>255.9794(4)	18-β-glycyrrhetinic acid
232	17.83#	384.2533	384.2525	-2.06	$C_{24}H_{33}NO_3$	MS^2[384]:<br>135.0437(100)<br>156.0646(43)<br>149.0593(21)<br>90.0547(15)		Guineensine
233	19.09#	227.2016	227.2010	-2.79	$C_{14}H_{28}O_2$		MS^2[227]:<br>227.2010(100)<br>180.8298(24)	Myristic acid
234	19.50#	342.2791	342.2784	-2.14	$C_{23}H_{35}NO$	MS^2[342]:<br>112.0756(100)<br>86.0967(50)<br>133.1009(14)<br>91.0545(27)		Dihydroevocarpine
235	19.75#	279.2329	279.2325	-1.37	$C_{18}H_{32}O_2$		MS^2[279]:<br>279.2326(100)<br>261.2222(43)<br>128.1673(15)<br>85.2074(5)	Linoleic acid
236	20.79#	355.1550	355.1549	-0.53	$C_{21}H_{24}O_5$		MS^2[355]:	Rutamarin

							116.9271(100)<br>115.9193(25)<br>100.9321(9)<br>84.9372(3)<br>99.9243(60)<br>355.1574(3)	
237	22.30#	336.3260	336.3252	-2.38	$C_{22}H_{41}NO$	MS^2[336]:<br>280.2627(14)<br>154.1221(7)<br>57.0705(65)<br>69.0704(33)<br>336.3251(100)		N-isobutyl-(2E,4E)-octadecadienamide

*Identified by comparison with standards. #First reported in Erdun-Uril
